# Clinical spectrum and molecular basis in 19 Chinese patients with 46, XY disorder of sexual development caused by *NR5A1* mutations

**DOI:** 10.1186/s13023-024-03472-8

**Published:** 2024-12-02

**Authors:** Yue Xu, Xuemeng Liu, Yang Liu, Hui Zhu, Jing Wu, Bing Han, Shiying Ling, Ren Cao, Haijun Yao, Yan Chen, Yu Liu, Yamin Rao, Xiaoyu Liu, Shuangxia Zhao, Huaidong Song, Jie Qiao

**Affiliations:** 1 Department of Endocrinology, Shanghai Ninth People’s Hospital, Shanghai Jiao Tong University School of Medicine, 639 Zhizaoju Road, Shanghai, 200011 China; 2 Department of Plastic and Reconstructive Surgery, Shanghai Ninth People’s Hospital, Shanghai Jiao Tong University School of Medicine, Shanghai, 200011 China; 3 The Core Laboratory in Medical Center of Clinical Research, State Key Laboratory of Medical Genomics, Department of Molecular Diagnostics and Endocrinology, Shanghai Ninth People’s Hospital, Shanghai Jiao Tong University School of Medicine, Shanghai, 200011 China; 4 Department of Urology, Shanghai Ninth People’s Hospital, Shanghai Jiao Tong University School of Medicine, Shanghai, 200011 China; 5 Department of Obstetrics and Gynecology, Shanghai Ninth People’s Hospital, Shanghai Jiao Tong University School of Medicine, Shanghai, 200011 China; 6 Department of Radiology, Shanghai Ninth People’s Hospital, Shanghai Jiao Tong University School of Medicine, Shanghai, 200011 China; 7 Department of Pathology, Shanghai Ninth People’s Hospital, Shanghai Jiao Tong University School of Medicine, Shanghai, 200011 China; 8 Department of Endocrinology and Metabolism, Huangpu Branch of Shanghai Ninth People’s Hospital, Shanghai Jiao Tong University School of Medicine, Shanghai, 200011 China

**Keywords:** Nuclear receptor subfamily 5 group A member 1 (*NR5A1*) mutation, Disorder/differences of sexual development (DSD), Genotype–phenotype correlation, Leydig cell development

## Abstract

**Background:**

Nuclear receptor subfamily 5 group A member 1 (*NR5A1*) plays pivotal roles in steroidogenesis and gonadal development. 46, XY disorder of sexual development (DSD) caused by *NR5A1* mutations is a rare genetic condition. This study aimed to provide a comprehensive analysis of the clinical characteristics and molecular defects observed in 19 Chinese patients with *NR5A1* variants, including assessing the deleterious effects of novel variants in vitro and evaluating their functional impact on the gonad and adrenal glands in vivo.

**Materials and methods:**

Subjects with *NR5A1* variants were identified from 223 Chinese 46, XY DSD patients via next-generation sequencing. In-silico analysis and functional assays were performed to evaluate the transcriptional activity, expression levels and nuclear localization of novel *NR5A1* variants. The histological structure of the gonads was evaluated via immunohistochemistry (IHC).

**Results:**

Patients with *NR5A1* gene variants presented with serious conditions, including micropenis, cryptorchidism, azoospermia, and radiological abnormalities of the spleen. Five novel *NR5A1* variants were identified, including heterozygous p.Y5*, p.Q42E and p.L359_L363del, as well as copy number variation (CNV) of chr9:127213317–127570245_del and an exon 6 duplication. A total of 63.2% (12/19) of patients harbored additional variants other than *NR5A1*. Defective transcriptional regulatory activities and abnormal protein expression levels were observed in *NR5A1* variants. The reduced levels of DHEA-S and 11-oxygenated steroids indicate a mild impairment in adrenal function among certain patients. The IHC analysis of the testis revealed intact expression levels of SOX9 in Sertoli cells, while significant differences were observed in the expression pattern of CYP17A1 in Leydig cells among patients. The preserved maturation of adult Leydig cells in the patients may trigger spontaneous puberty.

**Conclusions:**

Patients with *NR5A1* mutations exhibit complex phenotypes. The observed clinical heterogeneity may be attributed to oligogenic mutations, dysregulated Leydig cell function, as well as the impaired ability to modulate the transcription of target genes.

**Supplementary Information:**

The online version contains supplementary material available at 10.1186/s13023-024-03472-8.

## Background

Nuclear receptor subfamily 5 group A member 1 (NR5A1), also known as steroidogenic factor 1 [1] or adrenal 4-binding protein [2] (SF1 /Ad4BP), is an orphan nuclear receptor. It is encoded by the *NR5A1* gene which is located on 9q33.3 [3]. *NR5A1* has one noncoding exon and six coding exons and consists of 461 amino acids characterized by a DNA-binding domain (DBD) containing two zinc fingers and an FTZ-F1 box, an accessory hinge region, a ligand-binding domain (LBD) and two functional activation domains, AF-1 and AF-2 [4, 5]. The expression of *NR5A1* initiates in the urogenital ridge and subsequently becomes distributed across various tissues including the adrenal gland, gonad, ventromedial hypothalamus, pituitary gland and spleen. As a crucial transcription factor in steroidogenesis and reproductive development, *NR5A1* also exerts regulatory control over numerous genes involved in the hypothalamic–pituitary–adrenal and hypothalamic–pituitary–gonadal axes, thereby playing a pivotal role in orchestrating hormone profiles and reproduction [6].

Since the first *NR5A1* mutation was identified by Achermann et al. [7], over 188 mutations have been documented. The majority of these mutations were found in heterozygosity, with only 3% occurring in homozygosity [8]. Loss-of-function mutations in *NR5A1* correlate with a wide clinical spectrum of disorders of sex development (DSDs). In 46, XY males harboring *NR5A1* mutations, the presentation ranged from complete gonadal dysgenesis (CGD), partial gonadal dysgenesis (PGD) with micropenis, hypospadias and/or cryptorchid, to azoospermia without abnormal external genitalia development [9–16]. In 46, XX females, *NR5A1* mutations were revealed to be related to primary ovarian insufficiency (POI) [17, 18] and 46, XX testicular DSD/ovotesticular DSD (TDSD/OTDSD) [19–24]. Primary adrenal insufficiency was found in very rare patients with the homozygous mutation p.R92Q and the heterozygous mutations p.G35E, p.G35D and p.R255L [25, 26].

A wide range of clinical heterogeneity has been extensively discussed among individuals with *NR5A1* variants. Previous studies have revealed the absence of a phenotype‒genotype correlation in *NR5A1* mutations. Herein, we present a comprehensive analysis of the clinical characteristics and molecular basis of 19 Chinese 46, XY DSD patients with *NR5A1* variants identified through next-generation sequencing (NGS) from a cohort of 223 individuals—one of the largest cohorts among Chinese individuals. The in vitro functional alterations of novel *NR5A1* variants and histological analysis of patient gonads were also investigated to gain insights into the molecular mechanism underlying this disorder.

## Materials and methods

### Patients

This study enrolled 223 Chinese 46, XY DSD patients, who visited our hospital between 2009 and 2023. Male patients presented with variable degrees of undermasculinization, such as micropenis, hypospadias, bifid scrotum or cryptorchid. Patients with a female social gender presented with absence of breast development, primary amenorrhea, and inguinal cryptorchidism or pubertal virilization. All of their chromosome karyotypes were confirmed to be 46, XY. Patients who were clinically diagnosed with syndromic DSD and congenital adrenal hyperplasia were excluded.

This study was approved by the Ethics Committee of our hospital and written informed consent was obtained from the patients and/or their guardians for DNA collection to conduct molecular studies and research purposes.

### Next-generation sequencing and data analysis

Genomic DNA was extracted from peripheral blood samples via the QuickGene DNA Whole Blood Kit L (Kurabo, Japan) according to the manufacturer’s instructions. Genomic sequences and coding information were obtained from the UCSC and NCBI databases. In accordance with the period of patient recruitment, different types of genetic sequencing were used. Multiplex PCR was performed via the Access Array™ microfluidics platform (Fluidigm, USA), which contains 80 DSD-related genes [27], in 157 patients, whereas the probe-capture-targeted method (MyGenostics, China), which contains 281 DSD-related genes, was used in 54 patients. Thirteen patients underwent whole-exome sequencing. An Illumina HiSeq2500 system (Illumina, USA) was used for sequencing. Variants with a frequency < 1% were retained via the dbSNP135, ESP6500, 1000 Genomes Project, and ExAC databases to eliminate common single-nucleotide polymorphisms (SNPs). The variants were confirmed by Sanger sequencing. Nineteen patients with *NR5A1* mutations were identified and enrolled for further study.

### In silico analysis

The potential pathogenicity of the identified variants with respect to protein function was determined via in silico tools, such as CADD (Combined Annotation Dependent Depletion), Mutation Taster, Polyphen-2 and REVEL (rare exome variant ensemble learner) [28]. The clinical and genetic information of patients was used to classify the *NR5A1* variants. The classification was performed in accordance with the criteria of the American College of Medical Genetics and Genomics (ACMG) [29].

### Clinical data

The clinical data of patients were obtained from medical records, including the initial presentation (clinical, biochemical and radiological), treatment, and outcome. The External Masculinization Score (EMS) was used to evaluate external genitalia development. In prepubertal patients, the hCG stimulation test was introduced by intramuscular injection of 2000 IU chorionic gonadotrophin (Livzon Pharmaceutical Group Inc., China) for every other day with blood sampling of the serum testosterone level before injection (basal T) and 6 days after injection of hCG (post-hCG T). In prepubertal patients, post-hCG T increases five- to tenfold than basal T and has a concentration of > 110 ng/dl, indicating normal Leydig cell function [30]. Adrenocortical function was assessed by diurnal rhythms of ACTH and cortisol (8 am, 4 pm and midnight) without stimulation or rapid ACTH stimulation. 25U adrenocorticotropin (SPH No.1 Biochemical & Pharmaceutical Co., Ltd., China) was injected intravenously at 8 am and blood was collected before injection and at 30 min and 60 min postinjection. An 8 am cortisol or peak cortisol level above the threshold of 18 μg/dl (measured by the CLIA method) at either 30 or 60 min excludes adrenal insufficiency [31]. The levels of fourteen serum hormone, including four 11-oxygenated androgens, 11-ketotestosterone (11KT), 11β-hydroxytestosterone (11OHT), 11-ketoandrostenedione (11KA4) and 11β-hydroxyandrostenedione (11OHA4), were measured via LC‒MS/MS.

### In vitro functional assay

#### Plasmids

A wild-type *NR5A1* overexpression plasmid (pcDNA3.1-*NR5A1*-WT) and a luciferase reporter (pGL3-Basic-*CYP11A1* promoter and pGL3-Basic-*CYP19A1* promoter) were constructed as described [27]. Using pcDNA3.1-*NR5A1*-WT as a template, six mutant *NR5A1* overexpression vectors, including p.Y5*, p.Q42E, p.R84C, p.R313C, p.L359_L363del and p.Y404fs, were created via site-directed mutagenesis. A total of 1.6 kb of the human *AMH* promoter upstream of the transcriptional start site was amplified via PCR from the genomic DNA of HEK-293 T cells and inserted into the multiple cloning sites of pGL3-Basic, leading to the production of the pGL3-Basic-*AMH* promoter. The primers used are listed in Tables S1 and S2.

#### Luciferase reporter assay

293 T cells were plated in 24-well plates and cotransfected with the *NR5A1* wild-type or mutant overexpression plasmid (200 ng/well), firefly luciferase reporter (*CYP11A1* promoter, *CYP19A1* promoter or *AMH* promoter) (200 ng/well) or Renilla luciferase reporter pRL-TK (10 ng/well), which served as the internal reference, using Liposomal Transfection Reagent (Cat# 40802ES02, YEASEN, China). Luciferase activity was measured with a Dual-Luciferase Reporter Gene Assay Kit (Cat# 11402ES60, YEASEN, China) according to the manufacturer’s instructions. Firefly luciferase activity was normalized to Renilla luciferase activity and presented as relative luciferase activity. The samples were measured in triplicate in each assay, and all the assays were independently repeated more than three times.

#### Western blotting

The *NR5A1* overexpression plasmids of the wild-type or the mutants (pcDNA3.1-*NR5A1*-WT or pcDNA3.1-*NR5A1*-Mut) were transfected into 293 T cells, and whole-cell lysates were collected using RIPA lysis buffer (Cat# P0013B, Beyotime, China). After SDS‒PAGE, immunoblotting was performed with an anti-steroidogenic factor 1/STF-1 antibody (1:1000, Cat# 12800S, Cell Signaling Technology, USA) and an anti-GAPDH antibody (1:10,000, Cat# HA721136, HUABIO, China). Anti-rabbit IgG (1:2000, Cat# 7074, Cell Signaling Technology, USA) was used as a secondary antibody. The individual bands were measured with ImageJ software.

#### Subcellular localization analysis

The wild-type EGFP-tagged *NR5A1* plasmid (p.*NR5A1*-WT-EGFP-N2) was used as a template to generate four mutants (p.Q42E, p.R84C, p.R313C and p.L359_L363del). The primers used are presented in Table S1. The plasmids were transfected into 293 T cells seeded in a confocal dish with Liposomal Transfection Reagent. After 24 h, the medium was removed, and the cells were fixed with 4% paraformaldehyde fixative solution (Cat# P0099, Beyotime, China) for 15 min. Nuclear counterstaining was performed with 4,6-diamidino-2-phenylindole (DAPI) (Cat# C1005, Beyotime, China) for 5 min. Images were obtained via confocal laser scanning microscopy (Nikon, Japan).

### Sequence alignment and molecular modeling of NR5A1

Protein sequences were obtained from the NCBI database and uploaded to the UGENE program (Unipro UGENE, v.1.12.2), and multiple sequence alignment was performed. Three-dimensional protein structures were generated via AlphaFold v.2.3.0. PyMOL was used to observe and analyze the structural characteristics [32].

### Immunohistochemistry (IHC) and histology

Fixed gonadal tissues were embedded in paraffin, and blocks were cut to generate 4 μm sections. Deparaffinized sections were stained with either hematoxylin–eosin (HE) or antigen retrieval first, blocked and then incubated overnight at 4 °C with the primary antibodies anti-SOX9 (1:3000, Cat# ab185230, Abcam, USA) or anti-CYP17A1 (1:200, Cat# ab134910, Abcam, USA). The sections were incubated with an HRP-labeled secondary antibody (1:100, Cat# AS014, ABclonal, China) for 1 h at room temperature and IHC staining was detected with DAB (Cat# P0203, Beyotime, China). Images were captured by using a Nikon microscope (Nikon, Japan). A specially assigned doctor analyzed the pathological findings and determined the Johnsen score, which is an effective means of evaluating the histological features of the testes on a scale ranging from 1 to 10. A higher score represents a better status of spermatogenesis. Analysis of the IHC images was performed via ImageJ software.

### Statistical analysis

Statistical analyses were performed via GraphPad Prism 8.0 software (GraphPad Software, USA). The data are expressed as the means ± standard deviations (SDs). One-way ANOVA was used to determine the differences among multiple groups. *P* < 0.05 indicated a significant difference.

## Results

### Population characteristics

We identified 19 patients (8.5%) with *NR5A1* mutations among a total of 223 46, XY DSD patients, including 10 previously reported patients (I10-I19) [27] and 9 newly described patients (I1-I9). The ages of the 19 patients ranged from 1 to 28 years, with a median age of 15 years. The patients were distributed into different developmental stages, namely, childhood, adolescence and young adulthood, and included 8 prepubertal patients and 11 pubertal and postpubertal patients. Among the 19 patients, 11 patients were of the female social gender with chromosome karyotypes 46, XY, and 8 patients were of the male social gender (Table S3).

### Genetic analysis

**Fig. 1:**
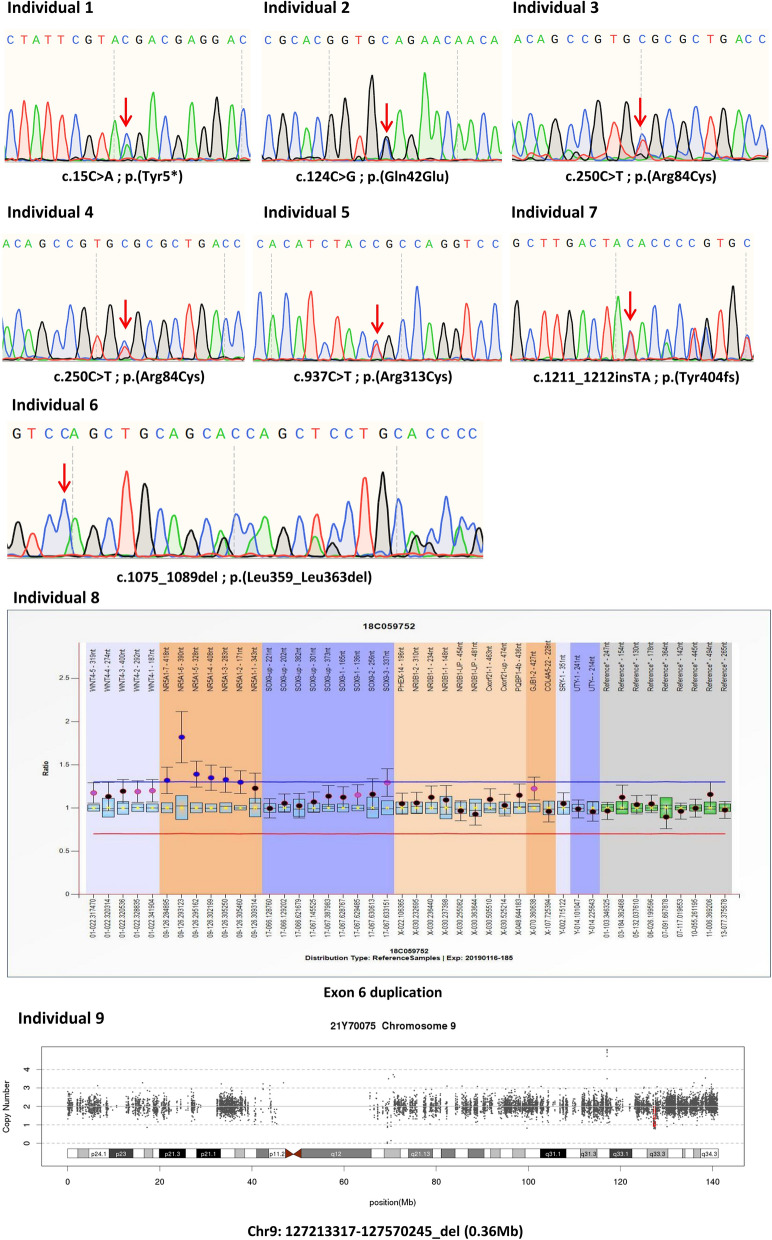
*NR5A1* heterozygous mutations detected in this study. c.15C > A; p.(Tyr5*) was identified in patient I1. c.124C > G; p.(Gln42Glu) was identified in patient I2. c.250C > T; p.(Arg84Cys) was identified in both patient I3 and patient I4. c.937C > T; p.(Arg313Cys) was identified in patient I5. c.1075_1089del; p.(Leu359_Leu363del) and c.1211_1212insTA; p.(Tyr404fs) were identified in patients I6 and I7, respectively. Patient I8 had an exon 6 duplication in *NR5A1*. Patient I9 had chromosome 9: 127,213,317-127570245_del (0.36 Mb del), containing *NR5A1*, *NR6A1*, *MIR181A2HG*, *MIR181A2*, *MIR181B2* and *OLFML2A*

Ten variants (6 missense: p.T29K, p.G35V, p.G212S, p.E367G, p.C370Y and p.S430I, 1 nonsense: p.C283*, 2 frameshift: p.R89fsX10 and p.E148fsX295 and 1 deletion: p.N44del) were identified in previously reported cases [27]. Eight variants, including 3 missense (p.Q42E, p.R84C and p.R313C), 1 nonsense (p.Y5*), 1 frameshift (p.Y404fs) and 3 indels (p.L359_L363del, exon 6 duplication and 0.36 Mb CNV chr9:127,213,317-127570245_del containing *NR5A1*, *NR6A1*, *MIR181A2HG*, *MIR181A2*, *MIR181B2* and *OLFML2A*) were identified in 9 new patients from 9 unrelated families (Fig. 1). p.R84C was found in both I3 and I4. According to the pedigree analysis (Fig. S1), the mutations identified in I1, I6, I8 and I9 were proven to be de novo. The p.Y5*, p.Q42E, p.L359_L363del, chr9:127,213,317-127570245_del (0.36 Mb) CNV and exon 6 duplication of *NR5A1* were novel mutations identified in this study for the first time. The p.R84C [33, 34], p.R313C [34–38] and p.Y404fs [39, 40] mutations have been reported in previous studies. Functional studies have also been performed on p.R84C and p.R313C, so they served as positive controls in the following functional studies. The mutation sites were distributed mainly in the DBD and LBD (Fig. S2). The pathogenicity of these variants, assessed according to the ACMG guidelines, is summarized in Table 1. Variants in other phenotypically associated genes carried by patients are listed in Table S4.

**Table 1:** NR5A1 mutations and other gene mutations identified in the cohort

No	*NR5A1* gene: Nucleotide/Amino Acid Alterations	Genomic position	In Silico Prediction Tools				ACMG Classification	Inheritance	Other gene mutations
			REVEL	Mutation Taster	PolyPhen2	CADD Score			
I1	c.15C > A /p.(Tyr5*)	chr9:127,265,660	-	Deleterious	–	34	P	de novo	–
I2	c.124C > G /p.(Gln42Glu)	chr9:127,265,478	0.822	Deleterious	Possibly damaging	25.2	VUS	NA	*LHCGR*: p.E243K (het)
I3	c.250C > T /p.(Arg84Cys)	chr9:127,262,989	0.948	Deleterious	Probably damaging	28.0	LP	NA	–
I4	c.250C > T /p.(Arg84Cys)	chr9:127,262,989	0.948	Deleterious	Probably damaging	28.0	LP	Mother: WT Father: NA	–
I5	c.937C > T /p.(Arg313Cys)	chr9:127,255,362	0.916	Deleterious	Probably damaging	27.7	P	NA	*MAMLD1*: p.P308L (hemi)
I6	c.1075_1089del /p.(Leu359_Leu363del)	chr9:127,253,409–127253423	–	Deleterious	–	–	LP	de novo	–
I7	c.1211_1212insTA /p.(Tyr404fs)	chr9:127,245,211–127,245,212	–	Deleterious	–	–	VUS	NA	–
I8	Exon 6 duplication	–	–	–	–	–	LP	NA	*INSL3*: p.D124E (het) *SRD5A2*: p.F96L (het)
I9	CNV chr9: 127,213,317-127570245_del(0.36 Mb)*	chr9: 127,213,317–127,570,245	–	–	–	–	P	de novo	*MYRF*: p.S985L (het)

*NA* not available, *P* Pathogenic, *VUS* variant of unknown significance, *LP* Likely Pathogenic, *WT* wild type, *REVEL* rare exome variant ensemble learner; Mutation Taster: https://www.genecascade.org/MutationTaster2021/; Polyphen-2: Polymorphism phenotype (http://genetics.bwh.harvard.edu/pph2/); CADD: Combined Annotation Dependent Depletion (https://cadd.gs.washington.edu/snv); *Chromosome 9: 127,213,317–127,570,245 contains *NR5A1*, *NR6A1*, *MIR181A2HG*, *MIR181A2*, *MIR181B2* and *OLFML2A*;het, heterozygous; hemi, hemizygous

### Clinical features

All 19 patients were normotensive, and had no clinical manifestations of adrenal insufficiency, such as skin pigmentation, nausea, vomiting or fatigue. These patients presented with variable severity of micropenis (18/19, 95%) and different types of hypospadias (18/19, 95%). The anatomical locations of the testes could be intrascrotal (10/36, 28%), inguinal (22/36, 61%) or abdominal (3/36, 8%). The EMS of patients reared as females was significantly lower than that of patients reared as males (*P* < 0.001). The clinical features of the 19 patients are presented in Tables S3 and S4. Two patients (I1 and I13) changed social gender from female to male and underwent cryptorchidopexy.

Sex hormones were analyzed in 15 patients; I2, I3 and I6 underwent surgery for cryptorchidectomy before the hormone assay and data of I12 were not available. The testicular function of 8 prepubertal patients was assessed via hCG stimulation tests. After hCG stimulation, the post-hCG T levels of four patients (I8, I14, I15 and I17) significantly increased by 6–61 times compared with the basal T, reaching the level of > 110 ng/dL. Conversely, the remaining four patients (I4, I5, I11 and I16) demonstrated deficiencies in testosterone biosynthesis. Among the 7 pubertal and postpubertal patients, 5 patients (I1, I7, I9, I13 and I19) presented normal levels of serum testosterone, and two patients (I10 and I18) presented lower levels. The level of testosterone was variable, with a median of 2.54 (0.15, 4.07) ng/mL. Notably, patient I10 presented significantly reduced testosterone levels, indicating severe gonadal dysgenesis. The levels of gonadotropic hormones were elevated in all 7 pubertal and postpubertal patients, indicating impaired negative feedback inhibition to the pituitary. The increase in the serum level of FSH was more prominent than that of LH. Moreover, 77% of patients (10/13) exhibited lower AMH levels than the reference range for the respective age groups.

Adrenal hormone levels were evaluated in 15 patients. The 8 am ACTH levels were elevated in 5/15 patients (I1, I5, I9, I14 and I17). Based on both the 8 am cortisol levels and cortisol levels following ACTH stimulation, 8 of the 15 patients showed no significant adrenal cortical dysfunction, while the remaining 7 did not meet the threshold, precluding a direct exclusion of adrenal cortical insufficiency. Among the 9 patients from whom samples were collected after adrenarche, 3 patients (I3, I7, and I9) had low DHEA-S levels. Notably, DHEA-S levels in patients I3 and I9 remained significantly below the age-related lower limits even after stimulation. The stimulated serum levels of P, 17-OHP and AD were found to be elevated to various degrees. The laboratory features of the 19 patients are presented in Tables S5, S6 and S7.

Considering the accuracy of the steroid assay, we assessed 14 steroids by LC‒MS/MS, including 11-oxygenated steroids, which could also reflect part of adrenal function. Our findings revealed that the median values of 11OHT, 11KA4 and 11OHA4 in 11 patients (postadrenarche age) were lower than the reference values reported by Davio et al.[41, 42], which were based on a cohort of 69 men aged between 18 and 39 years (Fig. S3 and Table S8). Notably, individual I9, who exhibited extremely low DHEA-S levels, also presented reduced levels of 11-oxygenated steroids, indicating inadequate production of adrenal-derived C19 steroids.

All patients had ultrasound or radiological abnormalities of the internal genitalia at diagnosis. Three of the six patients (I3, I9 and I10) had delayed bone age. Notably, 6/8 patients (I2, I4, I5, I9, I17 and I19) had abnormal development of the spleen. I2, I5, I9 and I19 all had a spleen characterized by anomalous morphology and size with strip-shaped calcifications. I4 and I17 had normal spleen sizes and morphologies with punctate calcifications. Specifically, abnormalities in spleen size and morphology were observed in 4 out of 6 cases, whereas calcifications were present in all 6 cases. I9 exhibited an atypical (32 HU) and centimetric nodular lesion on the right adrenal gland, with a reduced volume of the remaining adrenal gland. The radiological features and typical radiographs at diagnosis are shown in Table S9 and Fig. S4.

### In vitro functional assays of NR5A1

**Fig. 2:**
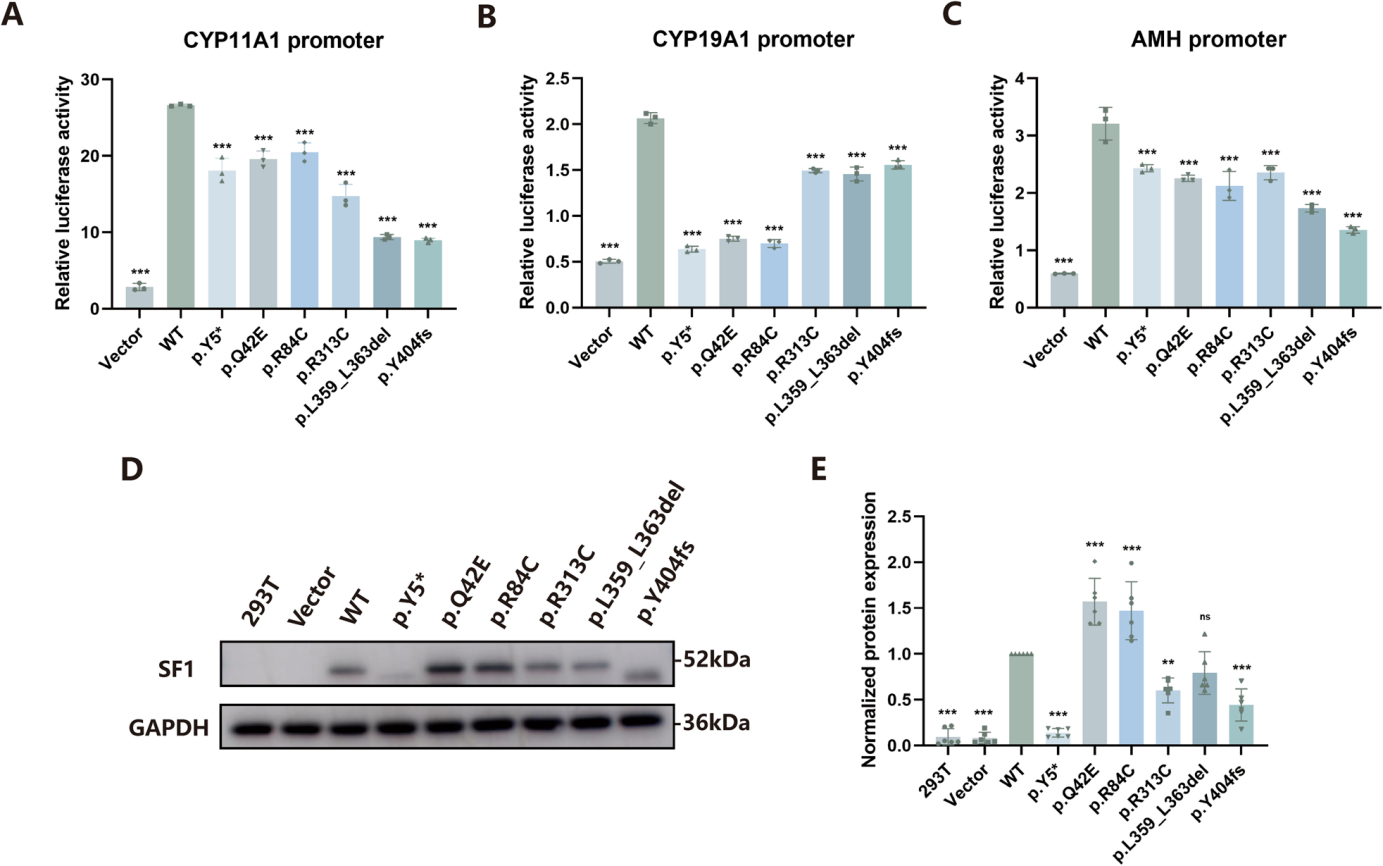
*NR5A1* gene transcriptional activity assays and protein expression analysis. (**A-C**) *NR5A1* gene transcriptional activity assays of WT NR5A1 and its variants. A luciferase reporter assay was used to assess the transcriptional activities of WT NR5A1 and its variants. Three promoter regions of the target genes (*CYP11A1*, *CYP19A1* and *AMH*) of *NR5A1* were included. (**D**) Western blot analysis of the protein expression levels of WT NR5A1 and its variants. Anti-GAPDH was used as a control. (**E**) Quantitative graph of the western blot analysis (D). One-way ANOVA was used. Data were represented as mean ± SD. ns, not significant, *P < 0.05, **P < 0.01, ***P < 0.001

Three promoter regions of the target genes (*CYP11A1*, *CYP19A1* and *AMH*) of *NR5A1* were included in the luciferase reporter assay. Compared with WT NR5A1, p.R84C and p.R313C have been reported to have decreased DNA-binding affinity and transcriptional activity [33, 35, 36], and they served as positive controls here. Compared with those in cells transfected with WT NR5A1, the variants constructed from 3 missense (p.Q42E, p.R84C and p.R313C), 1 frameshift (p.Y404fs), 1 nonsense (p.Y5*) and 1 deletion (p.L359_L363del) exhibited varying degrees of impaired transcription of the target gene (Fig. 2A–C).

Compared with that in cells transfected with WT NR5A1, the expression of normal NR5A1 was lower in cells transfected with p.Y5*, p.R313C and p.Y404fs variants. The protein expression of p.Y5* was comparable to that of the 293 T cells transfected with or without empty vectors. The expression of p.L359_L363del was comparable to that of the wild type. We also observed the expression of the truncated protein p.Y404fs, which has a lower protein molecular weight than the wild-type protein. Interestingly, the two variants p.Q42E and p.R84C resulted in increased protein expression levels (Fig. 2D, E).

Subcellular localization analysis was performed to assess the nuclear localization abilities of the four variants. As shown in Fig. S5, in p.EGFP-N2-vector-transfected cells, EGFP green fluorescence was distributed in the cytosol and nucleus, whereas EGFP-tagged WT NR5A1 was localized in the nucleus with a uniform distribution. Certain variants, such as the p.T29K and p.N44del variants, are known to have abnormal subnuclear localization, which showed nuclear localization with fine subnuclear puncta in our previous study [27]. The WT and p.R84C NR5A1 proteins were previously shown to be expressed at comparable levels and localized exclusively to the nucleus by Reuter et al. [33]. Compared with the abnormal variants, the variants p.Q42E, p.R313C and p.L359_L363del presented a nuclear localization pattern similar to that of WT NR5A1 and the positive control p.R84C. They did not alter trafficking or stability.

### Sequence alignment and molecular modeling

Multiple sequence alignments of the NR5A1 protein from various species revealed that R84 and R313 presented greater evolutionary conservation than Q42 (Fig. S6).

Three-dimensional front and dorsal views of the wild-type NR5A1 protein are shown in Fig. S7A and B. p.Y5* led to a premature stop codon, yielding a mere 4-amino-acid truncation (Fig. S7C, D). Compared with Q42, the E42 residue retained interactions (hydrogen bonds) with K38 (1.9 Å) and R87 (2.4 Å) but lost the interaction with R84 (1.9 Å) (Fig. S7E). The change from R84 (basic amino acid) to C84 (sulfur-containing amino acid) resulted in the loss of interaction with G90 (1.8 Å), G91 (1.9 Å), and R92 (2.4 Å), with only Q42 remaining as a bonding partner at a distance of 2.0 Å (Fig. S7F). R313 interacted with three amino acid residues, E237 (1.7 Å and 1.8 Å), D309 (2.1 Å) and H317 (2.0 Å), whereas C313 interacted with D309 (2.0 Å), H310 (2.9 Å), Q316 (2.8 Å) and H317 (2.0 Å) (Fig. S7G). In general, the p.Q42E, p.R84C, and p.R313C mutations resulted in a single amino acid substitution, thereby altering the local conformation of the wild-type protein. Conversely, the deletion of 5 amino acids (p.L359_L363del) led to a truncated α-helical structure with a disordered coil region. (Fig. S7H). A truncated protein of 429 amino acid residues was observed in p.Y404fs (Fig. S7I).

### Histological analysis of the gonads

**Fig. 3:**
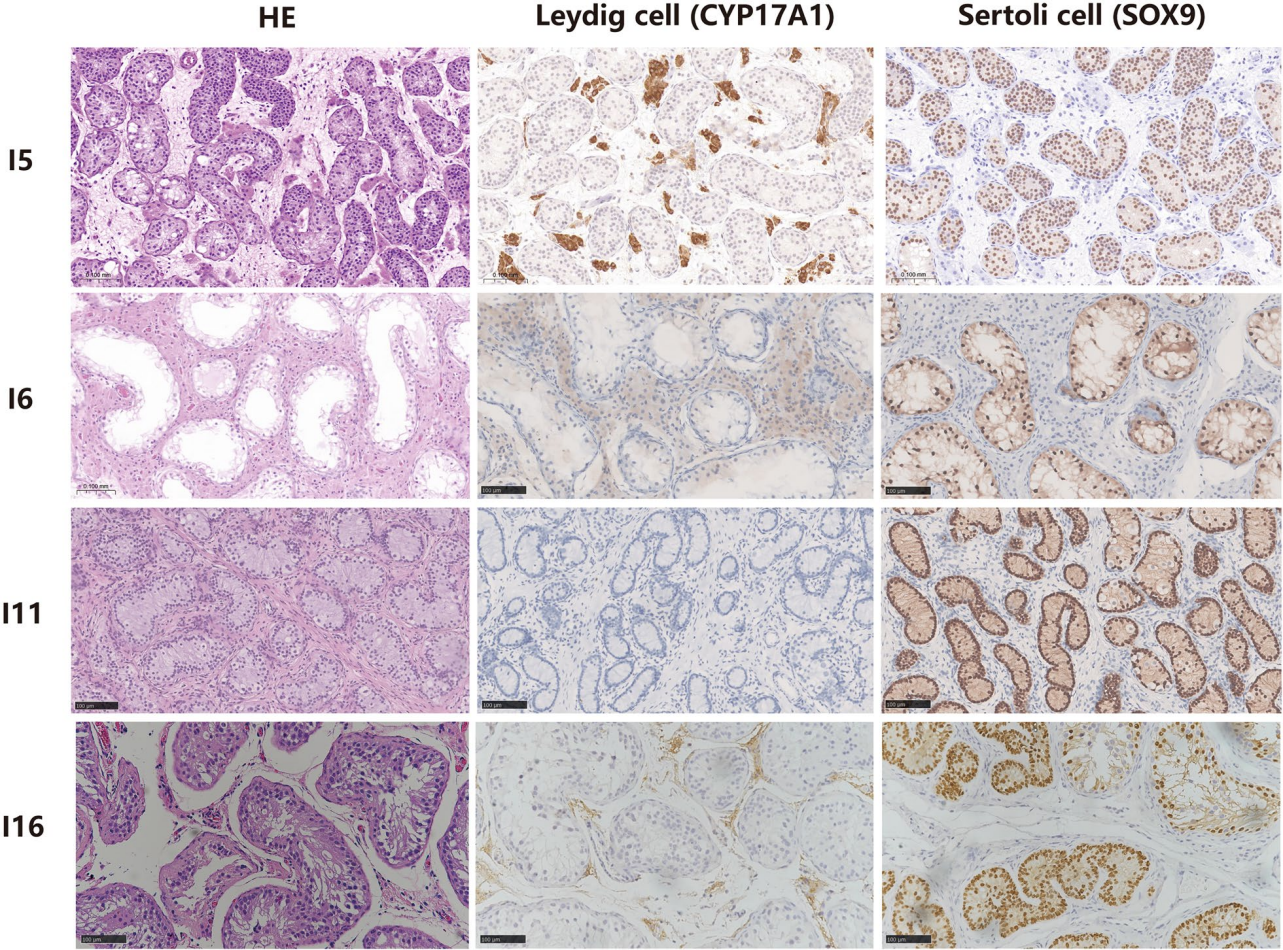
Histopathological testicular tissue analysis of patients with *NR5A1* mutations. Representative histologic images of testicular tissue from four typical patients (I5, I6, I11 and I16) are shown, while the histologic images of normal male testicular tissue are presented in Fig. S8. Pathological sections were stained with HE (left), and Leydig cells and Sertoli cells were positively stained by IHC with antibodies against CYP17A1 (middle) or SOX9 (right). Testicular seminiferous tubules were lined with Sertoli cells, and a few spermatogonia were observed. CYP17A1 ( +)-Leydig cell staining differed among patients (I5-29.2%, I6-45.7%, I11-2.9%, I16-10.7%), whereas there was little difference in SOX9 ( +)-Sertoli cell staining. Scale bar:100 μm

Eight patients (aged 5 to 22 years) underwent testicular biopsy (I1 and I16) or orchiectomy (I2, I5, I6, I7, I9, I11 and I16). They all presented with cryptorchidism, in which the testes were significantly reduced in size. In eight patients, histological examination via HE staining revealed the predominant presence of Sertoli cells, with a limited number of spermatogonia observed within the seminiferous tubules. A few spermatids were observed in two patients (I2, 18 years old; I7, 22 years old). Additionally, the absence of spermatozoa and Leydig cell hyperplasia were also noted. Both I2 and I7 had Johnsen scores of 6, whereas the remaining patients had scores of 3. Anti-SOX9 and anti-CYP17A1 antibodies were used to label Sertoli cells and Leydig cells, respectively. Notably, all eight subjects presented comparable expression levels of SOX9 in Sertoli cells. However, variations in CYP17A1-positive Leydig cell staining were observed among them. Specifically, the expression of CYP17A1 was significantly lower in two prepubertal patients (I11, 2.9% and I16, 10.7%) than in the other six individuals (I1, I2, I5, I6, I7 and I9, ranging from 19.0% to 46.3%). The HE and IHC results are listed in Table 2. Representative histologic images of typical patients and normal males are shown in Fig. 3 and Fig. S8.

**Table 2:** Histopathologic findings of the cohort

No	Age at surgery	HE staining				Johnsen Score	Anti-CYP17A1 IHC	T (ng/ml)		
		Spermatogenic cells	Sertoli cells	Leydig cells	Others			Pre-surgery		Post-surgery
								Pre- hCG	Post- hCG	
I1	15y	A few spermatogonia, no spermatozoa	Sertoli cells	A few Leydig cells hyperplasia	Degeneration and calcification were observed in some seminiferous tubules	3	19.0%	3.85	–	–
I2	18y	Spermatogonia, spermatids, no spermatozoa	Sertoli cells	Leydig cells hyperplasia	Focal fibrosis	6	46.3%	–	–	0.32↓
I5	10y	A few spermatogonia, no spermatozoa	Sertoli cells	–	–	3	29.2%	3.84	–	–
I6	15y	A few spermatogonia, no spermatozoa	Sertoli cells	–	–	3	45.7%	–	–	0.25↓
I7	22y	A few spermatogonia and spermatids, no spermatozoa	Sertoli cells	Leydig cells hyperplasia	–	6	21.0%	1.83	–	0.18↓
I9	17y	A few spermatogonia, no spermatozoa	Sertoli cells	Leydig cells hyperplasia	–	3	29.3%	4.07	–	–
I11	5y	A few spermatogonia, no spermatozoa	Sertoli cells	–	–	3	2.9%	0.08↓	0.16↓	–
I16	8y	A few spermatogonia and primary spermatocytes, no spermatozoa	Sertoli cells with focal hyperplasia	–	Small focal calcifications	3	10.7%	0.13↓	0.75↓	–

Quantification of CYP17A1-positive regions (cells) in testicular interstitial areas was performed using Fiji (ImageJ) software. The data are expressed as the mean values of at least six IHC images

*T* Testosterone, *y* years, ↓ below the reference range

T, 1.42–9.23 ng/ml

## Discussion

*NR5A1* is an important transcription factor in steroid synthesis, early sex determination and sex differentiation. *NR5A1* variants are considered one of the most common causes of 46, XY DSD, with a frequency of 8.5% (2/23) in Egyptian patients [43], 6.5% (5/77)−9% (9/100) in European patients [36, 44] and 8.6% (6/70) in Chinese patients [10], which was reported in several studies and confirmed as 8.5% (19/223) in our study. The wide and intricate phenotypic spectrum of *NR5A1* variants has been documented [45]. In this study, we sought to observe phenotype‒genotype associations and explore the underlying mechanism of phenotypic heterogeneity.

The clinical manifestations observed in these 19 patients included a range of phenotypes, including gonadal dysgenesis, undermasculinized external genitalia, micropenis, cryptorchidism, and hypospadias. Additionally, six patients exhibited an uncommon phenotype characterized by abnormalities in the spleen. Importantly, despite the absence of apparent adrenocortical insufficiency in these patients, some individuals were observed unresponsive cortisol, reduced DHEA-S levels or 11-oxygenated androgens. Within the cohort of 19 patients harboring various mutations, significant variations in clinical severity, EMS, and sex hormone levels were identified.

We also discovered that, in addition to the prevailing impairment of Sertoli cell function, the varying degrees of functional impairment of Leydig cells may play a more pivotal role in the observed phenotypic heterogeneity in PGD patients with *NR5A1* mutations. Specifically, the majority of patients in this cohort were diagnosed with 46, XY PGD characterized by regression of Müllerian duct structures, as evidenced by the absence of the uterus, fallopian tubes, and upper one-third of the vagina. These findings suggest that, during embryonic development, adequate secretion of AMH is capable of inducing regression of the Müllerian ducts, whereas insufficient testosterone secretion likely hinders complete external genitalia development. Among the 7 pubertal and postpubertal patients, 5 patients (EMS: 1 to 5.5) presented normal testosterone levels, whereas 2 patients (EMS: 1 and 5.5) presented reduced testosterone levels. Even if patients exhibit severe DSD phenotype with low EMS score, they may still have preserved adult Leydig cell function, which can trigger pubertal development within the normal age range. The findings validate the typical pubertal development observed in a subset (26%, 12/47) of individuals with *NR5A1* variants as reported by Kouri et al. [45]. Due to the differential origin of fetal Leydig cells and adult Leydig cells, the observed disparities in serum testosterone levels, coupled with varying degrees of external genital development, suggest that distinct variants of *NR5A1* exert diverse influences on the developmental stages of Leydig cells [46–48].

Histological alterations in the testes, especially the development of Sertoli cells and Leydig cells, were further investigated in patients. The expression of CYP17A1 (Leydig cells) varied among the eight patients, indicating variations in the development of Leydig cells among patients. Specifically, the expression of CYP17A1 in the Leydig cells of patients I11 (2.9%) and I16 (10.7%) was significantly reduced, which is consistent with the low levels of testosterone and unresponsive testicular function. However, patients I1, I5, I7 and I9 exhibited basal testosterone levels within the normal range, consistent with approximately 19.0–46.3% of Leydig cells positive for CYP17A1 in the testicular interstitium. This suggests that the phenotypic heterogeneity observed in these patients may be primarily attributed to aberrant Leydig cell development.

The prevalence of spleen anomalies in our cohort was observed in 6/8 patients, accounting for 75%, which aligns with the relatively frequent occurrence of organ defects. However, it has been reported that adrenal insufficiency is uncommon among individuals with *NR5A1* variants [8]. In our study, low 8 am or responsive cortisol levels were observed in 47% (7/15) of the patients, suggesting mild defects in adrenal reserve function. Reduced levels of DHEA-S were also observed in I3, I7, and I9, with I3 and I9 showing low levels even after stimulation during postadrenarche, indicating an impairment in the adrenal zona reticularis or a defect in 17,20-lyase activity. Furthermore, for the first time, the observed decrease in the levels of adrenal-derived 11-oxygenated steroids revealed a tangible impairment of adrenal function in specific patients with *NR5A1* mutations.

We also performed a series of in vitro experiments aiming to evaluate the functional discrepancies arising from different mutations. The mutation p.Q42E has been previously reported in the context of single-cell sequencing of the epididymis in patient I2 [49]. The p.R84C mutation was initially discovered in a 46, XY Japanese patient who exhibited mild gonadal dysgenesis but no adrenal insufficiency [33]. In the present study, the p.R84C mutation was identified in both patients I3 and I4. Interestingly, the p.Q42E and p.R84C variants were found to affect the transcriptional activation of target genes, despite not causing a reduction in the protein level of NR5A1. In addition, the p.R313C mutation has been extensively reported [35–37] and confirmed as a prevalent mutation in the Chinese population [34]. The mutations have varying effects on the transcriptional activity of target genes (CYP11A1, CYP19A1, and AMH), leading to distinct impairments in Leydig cells and Sertoli cells. This difference in cellular differentiation may contribute to the clinical phenotypic diversity.

Among the 19 patients included, 12 presented at least one additional gene mutation associated with abnormal sexual development. The presence of digenic/polygenic/oligogenic mutations may contribute significantly to the heterogeneity observed in the clinical phenotypes of 46, XY DSD patients [50]. Notably, 0.36 Mb del CNV was identified in patient I9, indicating a dose-dependent effect or haploinsufficiency of *NR5A1* variants. In fact, the genetic network comprising NR5A1/SF-1 is complex and includes transcription factors, coregulators, posttranslational modulators and signaling molecules. The 3D structure of the chromosome, encompassed by the active transcription factor *NR5A1*, remains unexplored during gonad development and steroidogenesis. In the future, by employing patient-specific induced pluripotent stem cell technology and targeted differentiation techniques [51], the underlying mechanism of the phenotypic heterogeneity of the patients could be elucidated.

In this study, we provide the clinical and molecular data of 19 Chinese 46, XY DSD patients harboring heterozygous *NR5A1* mutations. The novel mutations were identified, and subsequent in silico analysis, mutagenesis experiments, and the transactivation functions of the variants were conducted to demonstrate their deleterious effects. Additionally, comprehensive steroid profiles, including ACTH stimulation tests and 11-oxo-steroids, as well as histological features of the gonads, represent the in vivo functional impacts of *NR5A1* variants.

## Supplementary Information

Additional file1

## Data Availability

Supplemental materials cited in this work can be accessed at https://doi.org/10.6084/m9.figshare.27635697.v1. Other data supporting the findings of this study are not openly available due to reasons of sensitivity and to protect the privacy of individuals; however, data are available from the corresponding author upon reasonable request.

## Abbreviations

NR5A1: Nuclear receptor subfamily 5 group A member 1
DSD: Disorder/differences of sexual development
EMS: External masculinization score
NGS: Next-generation sequencing
AMH: Anti-Müllerian hormone
CNV: Copy number variation
DBD: DNA-binding domain
LBD: Ligand-binding domain
CGD: Complete gonadal dysgenesis
PGD: Partial gonadal dysgenesis
POI: Primary ovarian insufficiency
TDSD/OTDSD: Testicular DSD/ovotesticular DSD
hCG: Human chorionic gonadotropin
CAH: Congenital adrenal hyperplasia
SNP: Single-nucleotide polymorphism
ACMG: American College of Medical Genetics and Genomics
HEK: Human embryonic kidney
ACTH: Adrenocorticotropic hormone
AD: Androstenedione
FSH: Follicle-stimulating hormone
LH: Luteinizing hormone
T: Testosterone
IHC: Immunohistochemistry
LC–MS/MS: Liquid chromatography-tandem mass spectrometry
11KT: 11-Ketotestosterone
11OHT: 11β-Hydroxytestosterone
11KA4: 11-Ketoandrostenedione
11OHA4: 11β-Hydroxyandrostenedione
