# Supplementary material for: Clinical spectrum and molecular basis in 19 Chinese patients with 46, XY disorder of sexual development caused by NR5A1 mutations
Source: Orphanet J Rare Dis. 2024 Dec 2;19:453. doi: 10.1186/s13023-024-03472-8 (PMC11610102; doi:10.1186/s13023-024-03472-8)
Supplement: Supplementary file 1 — Additional file1 [file 13023_2024_3472_MOESM1_ESM.docx]

**Clinical spectrum and molecular basis in 19 Chinese patients with 46, XY disorder of sexual development caused by *NR5A1* mutations**

**Supplementary Materials**


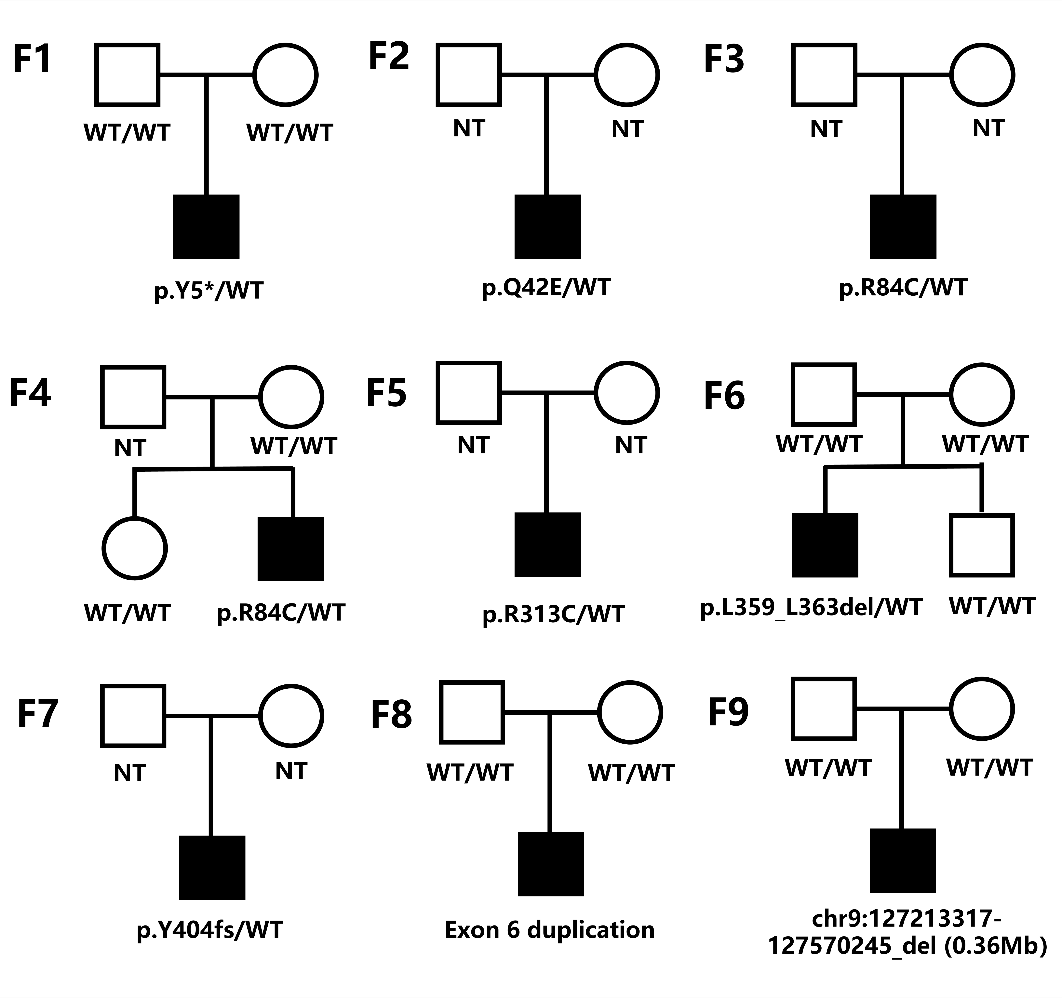


**Supplementary Figure 1. The pedigree tree of nine families newly reported in this study.**

NT indicates that the genotype was not tested in this family member.





**Supplementary Figure 2.** **Structure of NR5A1 protein and distribution of *NR5A1* mutations.**

The identified *NR5A1* mutations were mainly distributed in DBD and LBD.





**Supplementary Figure 3. Circulating concentrations of four 11-oxygenated androgens in eleven patients with *NR5A1* mutations.**

The median values of three 11-oxygenated androgens (11OHT, 11KA4 and 11OHA4) of eleven patients (post-adrenarche age) with *NR5A1* mutations were lower than the reference values (colored in grey) which were sourced from the data of 69 men aged 18-39 (Davio *et al*. 2020).


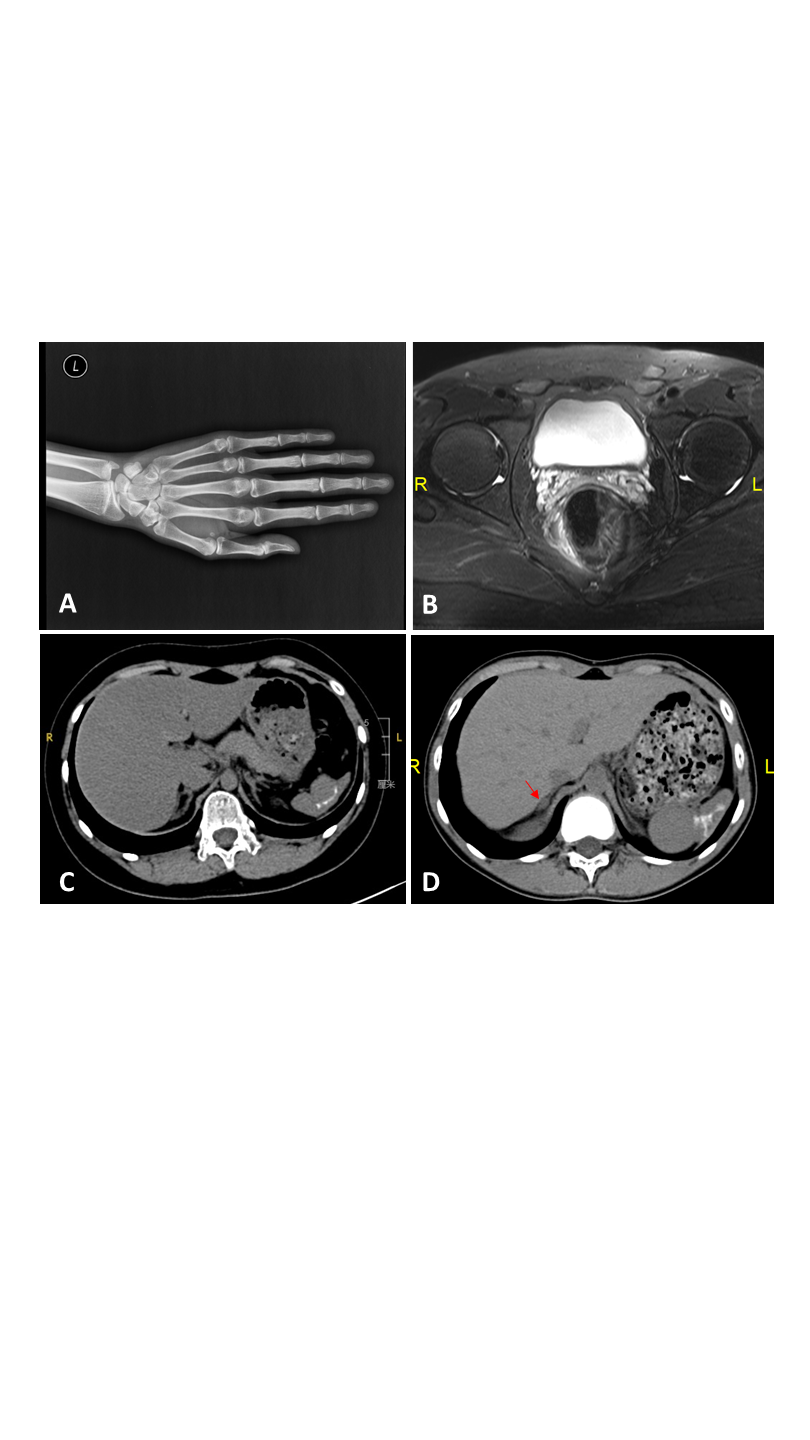


**Supplementary Figure 4.** **Radiological features of patients.**

**(A)** Bone age of patient I3 (chronological age: 24 years old) was 14 years old. **(B)** On axial T2WIfs, patient I7 showed bilateral ovaries loss and cryptorchidism in bilateral inguinal regions. **(C)** Patient I2 had a spleen characterized by anomalous morphology and small size with strip-shaped calcifications. **(D)** The spleen of patient I9 also exhibited anomalous morphology, characterized by the presence of strip-shaped calcifications and multiple nodules with the largest measuring approximately 4cm in diameter. A small nodular lesion was observed on the right adrenal gland, measuring approximately 7 × 4 × 10 mm (HU values: Min: 19, Max: 42, Avg: 32) (red arrow), with a reduced volume of the remaining adrenal gland.


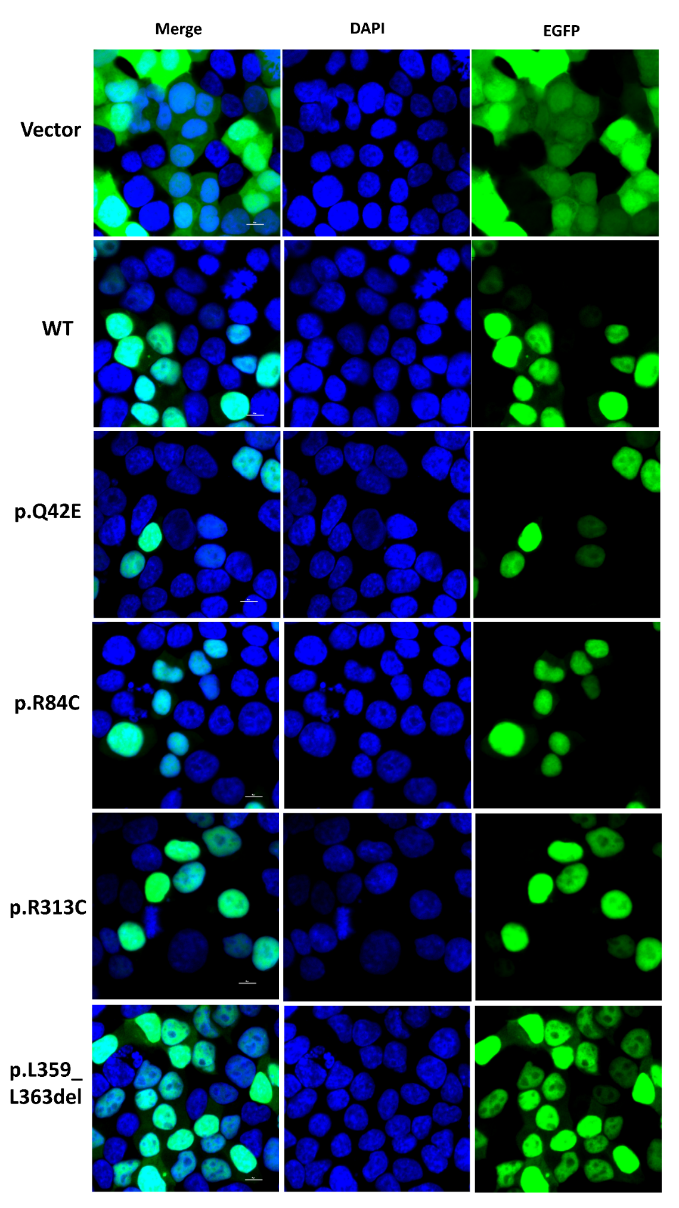


**Supplementary Figure 5. Subcellular localization analysis of WT NR5A1 and variants.**

EGFP green fluorescence was distributed to the cytoplasm and the nucleus in empty vector-transfected cells. EGFP-tagged WT NR5A1 showed nuclear localization with a uniform distribution. A similar nuclear localization pattern was observed for p.Q42E, p.R84C, p.R313C and p.L359_L363del. Scale bar:10μm.


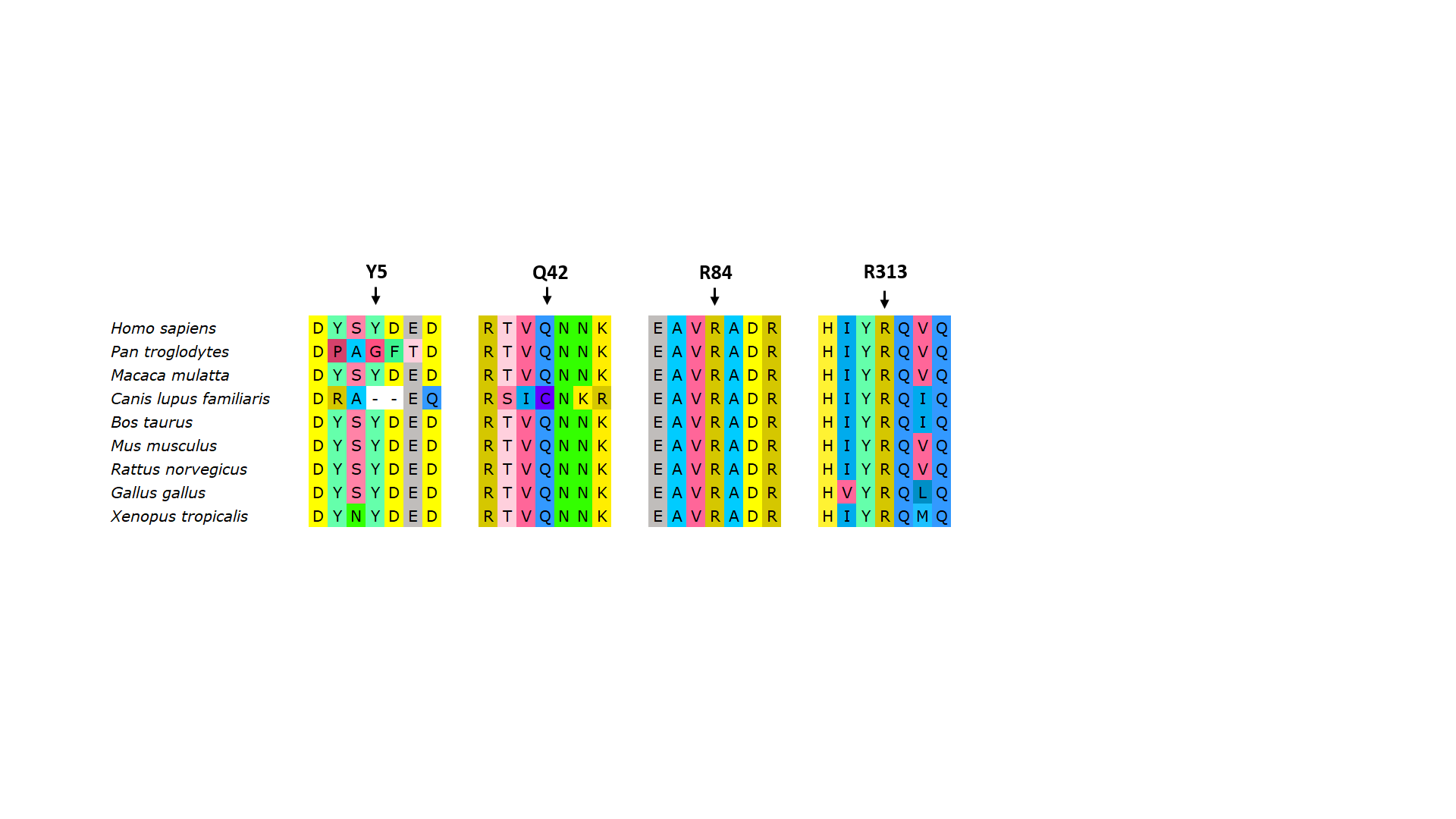


**Supplementary Figure 6. Multiple sequence alignments for the NR5A1 protein from various species.**

R84 and R313 exhibited higher evolutionary conservation than Q42.

**
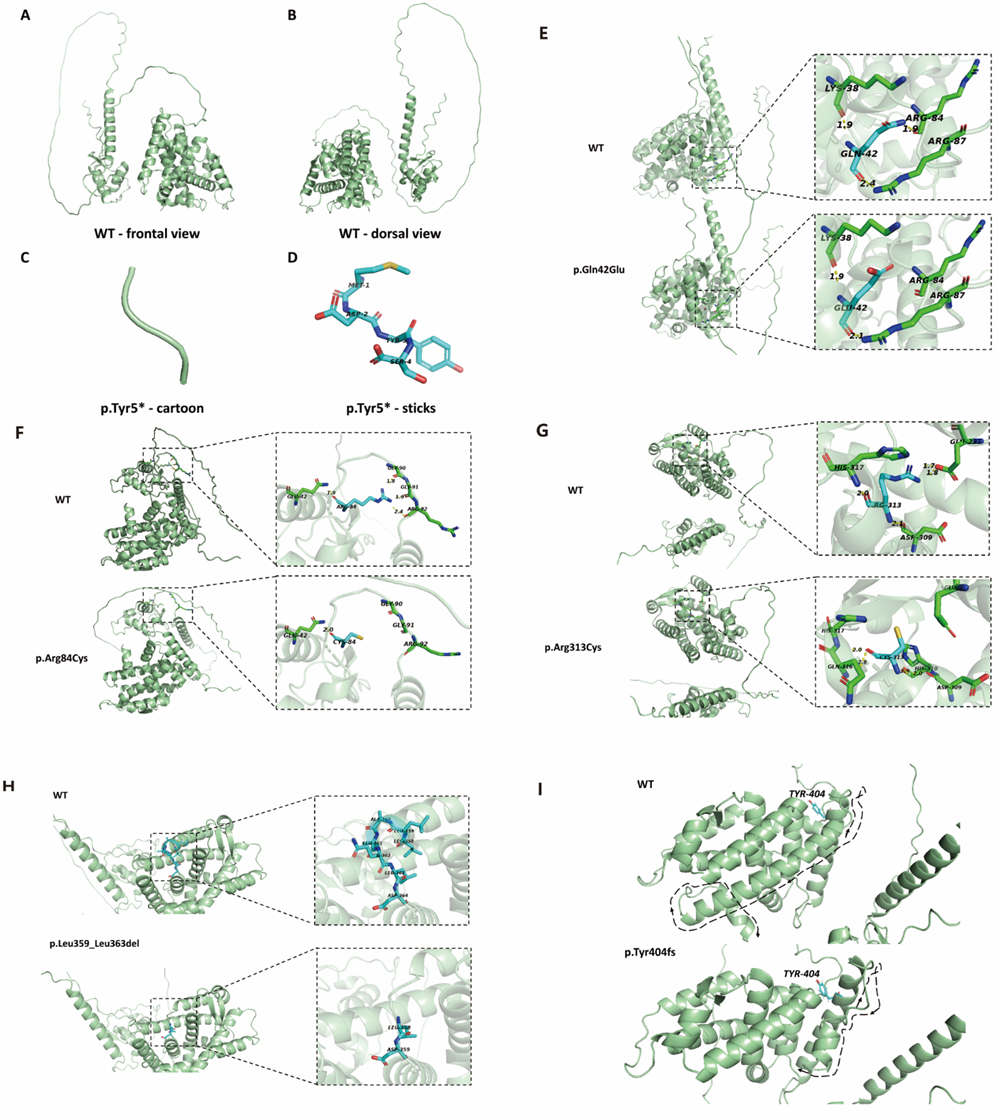
**

**Supplementary Figure 7. Molecular modeling of WT NR5A1 and its variants.**

**(A, B)** Three-dimensional (3D) front and dorsal views of the WT NR5A1 protein are shown. **(C, D)** The substitution of cytosine with adenine at nucleotide 15 within the coding region caused a conversion of the amino acid tyrosine to a stop codon at amino acid residue 5, resulting in the premature termination of protein translation. This alteration led to the truncation of only four amino acids. **(E)** Nucleotide substitution of cytosine for guanine at position 124 within the coding region led to the substitution of glutamine for glutamic acid at amino acid position 42 within the translated protein. Analysis of the 3D structure revealed that the Q42 residue could interact with three amino acid residues, namely K38 (1.9 Å), R84 (1.9 Å), and R87 (2.4 Å). Conversely, the E42 residue maintain interactions with K38 (1.9 Å) and R87 (2.4 Å), but lost its interaction with R84. **(F)** The substitution of cytosine with thymine at the 250th nucleotide of the coding region led to a change in the 84th amino acid residue from arginine (basic amino acid) to cysteine (sulfur-containing amino acid) in the resulting translated protein. Analysis of the protein's 3D structure revealed that the original arginine residue at position 84 was capable of forming hydrogen bonds with four adjacent amino acid residues: Q42 (1.9 Å), G90 (1.8 Å), G91 (1.9 Å), and R92 (2.4 Å). However, the substitution to cysteine at position 84 resulted in the loss of interaction with the latter three amino acid residues, with only Q42 remaining as a bonding partner at a distance of 2.0 Å. **(G)** The mutation of nucleotide 937 from cytosine to thymidine in the coding region resulted in a change in amino acid residue 313 from arginine (basic amino acid) to cysteine (sulfur-containing amino acid) in the translation product. The 3D structure showed that R313 interacted with three amino acid residues E237 (1.7 Å and 1.8 Å), D309 (2.1 Å) and H317 (2.0 Å), whereas C313 interacted with D309 (2.0 Å), H310 (2.9 Å), Q316 (2.8 Å) and H317 (2.0 Å). **(H)** A deletion of nucleotides spanning from 1075 to 1089 within the coding region led to the removal of five amino acids (Leu359-Ala360-Leu361-Gln362-Leu363) from the translation product. This mutation caused a reduction in the number of helical turns within the α-helix structure, decreasing from five to three. **(I)** Two nucleotides (thymine and adenine) were inserted between the 1211 to 1212 nucleotides of the coding region, which resulted in a change in the amino acid residue after the tyrosine at amino acid position 404 of the translation product. Amino acid position 430 became a stop codon, which caused premature termination of protein translation. The structure changed greatly after mutation, and the first two α-helix structures were replaced by a smaller α-helix structure (the black dotted line arrow indicates the structure of amino acid residues before and after mutation).


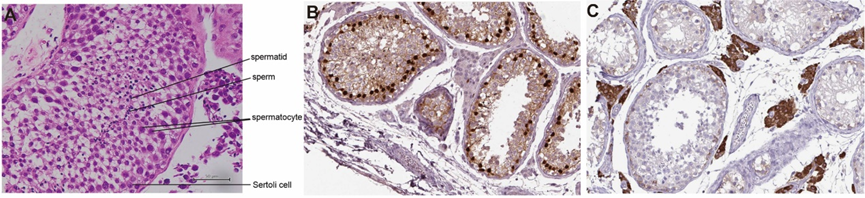


**Supplementary Figure 8. Histopathological testicular tissue analysis of healthy males.**

**(A)** Pathological image for JSC 8–10. The image is stained with haematoxylin eosin and ×400 magnification. JSC 8–10 include few or many sperms in a seminiferous tubule. From Ito Y, Unagami M, Yamabe F, et al. A method for utilizing automated machine learning for histopathological classification of testis based on Johnsen scores. Sci Rep. 2021;11(1):9962. Published 2021 May 10. doi:10.1038/s41598-021-89369-z.[1]

**(B)** The pathological image is derived from the normal testicular tissue of a 29-year-old male. Sertoli cells were positively stained by IHC with antibody against SOX9. Male, age 29, Testis, Normal tissue.

From website: Tissue expression of SOX9 - Staining in testis - The Human Protein Atlas

(<https://www.proteinatlas.org/ENSG00000125398-SOX9/tissue/testis>).

**(C)** The pathological image is derived from the normal testicular tissue of a 29-year-old male. Leydig cells were positively stained by IHC with antibody against CYP17A1. Male, age 29, Testis, Normal tissue.

From website: Tissue expression of CYP17A1 - Staining in testis - The Human Protein Atlas

(<https://www.proteinatlas.org/ENSG00000148795-CYP17A1/tissue/testis>).

**Supplementary Table 1. Primers for mutant plasmids construction**

| **Mutation** | **Forward (5’-3’)** | **Reverse (5’-3’)** |
| --- | --- | --- |
| p. Y5* | CATGGACTATTCGTAAGACGAGGACCT | TTACGAATAGTCCATGAATTCTGCAGAT |
| p. Q42E | TTCAAGCGCACGGTGGAGAACAACAAG | CCACCGTGCGCTTGAAGAAGCCCTTGC |
| p. R84C | GCGCCTGGAAGCCGTGTGCGCTGACCG | ACACGGCTTCCAGGCGCATCCCCACCG |
| p. R313C | TTCGACCACATCTACTGCCAGGTCCAG | AGTAGATGTGGTCGAACACCAGCAGCTC |
| p. L359_L363del | CTGGTGCTGCAGCTGGACCGGCAGGAG | CCAGCTGCAGCACCAGCTCCTGCGCC |
| p. Y404fs | CCGCCCTGCTTGACTATACACCCTGTGCC | TATAGTCAAGCAGGGCGGCGTTGGCCTTC |

pcDNA 3.1-h*NR5A1*-WT and p.h*NR5A1*-WT-EGFP-N2 was used as plasmid template.

**Supplementary Table 2. Primers for pGL3-Basic-*AMH* promoter**

|  | **Primers (5’-3’)** |
| --- | --- |
| pGL3-Basic-*AMH* promoter-Forward | TCTTACGCGTGCTAGCCCGGGGCCCTAGCCCCACCCGTG |
| pGL3-Basic-*AMH* promoter-Reverse | CAGTACCGGAATGCCAAGCTTGCCAGGGGCTGGGCTGCC |

**Supplementary Table 3. Demographic and clinical characteristics of the cohort**

| Variable (n) | n | % | Patient ID |
| --- | --- | --- | --- |
| Social gender (n=19) |  |  |  |
| Female | 11 → 9 | 58 → 47 | I2, I3, I5, I6, I7, I9, I10, I11, I14 |
| Male | 8 → 10 | 42 → 53 | I1*, I4, I8, I12, I13*, I15, I16, I17, I18, I19 |
| Age at diagnosis (n=19) |  |  |  |
| Prepubertal (<14y) | 8 | 42 | I4, I5, I8, I11, I14, I15, I16, I17 |
| Pubertal and post-pubertal (≥14y) | 11 | 58 | I1, I2, I3, I6, I7, I9, I10, I12, I13, I18, I19 |
| Symptoms at diagnosis (n=19) |  |  |  |
| External genitalia (n=19) |  |  |  |
| Male external genitalia | 0 | 0 | - |
| Ambiguous external genitalia  (Micropenis/Clitoris hypertrophy) | 18 | 95 | I1, I2, I3, I4, I5, I6, I7, I8, I9, I11, I12, I13, I14, I15, I16, I17, I18, I19 |
| Female external genitalia | 1 | 5 | I10 |
| Hypospadias (n=18) |  |  |  |
| Mild (distal penile) | 0 | 0 | - |
| Moderate (mid-penile) | 3 | 17 | I3, I4, I12 |
| Severe (proximal penile) | 15 | 83 | I1, I2, I5, I6, I7, I8, I9, I11, I13, I14, I15, I16, I17, I18, I19 |
| Testes/Cryptorchid (n=18×2=36) |  |  |  |
| Absent | 1 | 3 | I2 |
| Abdominal | 3 | 8 | I1, I9 |
| Inguinal | 22 | 61 | I2, I3, I5, I6, I7, I9, I11, I12, I13, I14, I16, I17, I18, I19 |
| Intrascrotal | 10 | 28 | I4, I8, I13, I15, I17, I18, I19 |
| Bifid scrotum (n=19) |  |  |  |
| Yes | 11 | 58 | I1, I2, I3, I5, I6, I7, I9, I11, I13, I14, I15 |
| No | 8 | 42 | I4, I8, I10, I12, I16, I17, I18, I19 |
| Amenorrhea (n=8) |  |  |  |
| Yes | 8 | 100 | I1, I2, I3, I6, I7, I9, I10, II13 |
| No | 0 | 0 | - |
| Voice change during puberty (n=11) |  |  |  |
| Yes | 8 | 73 | I1, I2, I6, I7, I9, I13, I18, I19 |
| No | 3 | 27 | I3, I10, I12 |

*After a definitive diagnosis, 2 patients (I1 and I13) changed social gender from female to male.

**Supplementary Table 4. Clinical and genetic findings of the cohort**

| **No.** | **Age** | **Gender** | **Phenotype** | | | | | | | | **EMS** | **Surgery** | ***NR5A1* mutations** | **Other genes mutations** |
| --- | --- | --- | --- | --- | --- | --- | --- | --- | --- | --- | --- | --- | --- | --- |
|  |  |  | **Micropenis** | **Hypo-**  **spadias** | **Testes/Cryptorchid** | | **Bifid scrotum** | **Amenorrhea** | **Voice change during puberty** | **Others** |  |  |  |  |
|  |  |  |  |  | **Left** | **Right** |  |  |  |  |  |  |  |  |
| **I1** | 15y | F→M | √ | perineal | abdominal | abdominal | √ | √ | √ | × | 1 | cryptorchidopexy | p.Y5* (het) | × |
| **I2** | 19y | F | √ | perineal | inguinal | × | √ | √ | √ | abnormal spleen | 1 | cryptorchidectomy | p.Q42E (het) | *LHCGR*: p.E243K (het) |
| **I3** | 24y | F | √ | mid-penile | inguinal | inguinal | √ | √ | × | delayed bone age | 3 | cryptorchidectomy | p.R84C (het) | × |
| **I4** | 10y | M | √ | mid-penile | intrascrotal | intrascrotal | × | / | / | abnormal spleen, small palpebral fissure | 7 | hypospadias repair | p.R84C (het) | × |
| **I5** | 5y | F | √ | perineal | inguinal | inguinal | √ | / | / | abnormal spleen | 2 | cryptorchidectomy | p.R313C (het) | *MAMLD1*: p.P308L (hemi) |
| **I6** | 16y | F | √ | perineal | inguinal | inguinal | √ | √ | √ | × | 2 | cryptorchidectomy | p.L359_L363del (het) | × |
| **I7** | 22y | F | √ | perineal | inguinal | inguinal | √ | √ | √ | × | 2 | cryptorchidectomy | p.Y404fs (het) | × |
| **I8** | 1y | M | √ | perineal | intrascrotal | intrascrotal | × | / | / | × | 6 | hypospadias repair | Exon 6 duplication | *INSL3*: p.D124E (het) *SRD5A2*: p.F96L (het) |
| **I9** | 15y | F | √ | perineal | inguinal | abdominal | √ | √ | √ | abnormal spleen,  abnormal adrenal gland, delayed bone age | 1.5 | cryptorchidectomy | 0.36Mb del CNV | *MYRF*: p.S985L (het) |
| **I10** | 22y | F | × | × | undetected | undetected | × | √ | × | infantile uterus, FEG, vulvar dysplasia, delayed bone age | 1 | mammaplasty | p.G212S (het) | *SRY*: p.R76L (hemi)  *FGF10*: p.M204V (het) *SOX9*:enhancer del |
| **I11** | 5y | F | √ | perineal | inguinal | inguinal | √ | / | / | × | 2 | cryptorchidectomy | p.N44del (het) | × |
| **I12** | 21y | M | √ | mid-penile | inguinal | inguinal | × | / | × | × | 6 | × | p.C283* (het) | *CST9*: p.R87* (het) |
| **I13** | 19y | F→M | √ | perineal | inguinal | intrascrotal | √ | √ | √ | × | 2.5 | hypospadias repair cryptorchidopexy penis reconstruction | p.T29K (het) | *AR*: p.L295P (hemi) *MYH6*: p.A1411T (het) |
| **I14** | 3y | F | √ | perineal | inguinal | inguinal | √ | / | / | × | 2 | × | p.E148fsX295 (het) | × |
| **I15** | 4y | M | √ | perineal | intrascrotal | intrascrotal | √ | / | / | × | 3 | hypospadias repair penis reconstruction | p.R89fsX10 (het) | *EGF*: p.V999M (het) |
| **I16** | 8y | M | √ | perineal | inguinal | inguinal | × | / | / | × | 5 | cryptorchidism needle biopsy | p.G35V (het) | *BMP2*: p.A106T (het) |
| **I17** | 3y | M | √ | perineal | inguinal | intrascrotal | × | / | / | abnormal spleen | 5.5 | hypospadias repair | p.C370Y (het) | *SOX3*: p.V53L (hom) |
| **I18** | 28y | M | √ | perineal | inguinal | intrascrotal | × | / | √ | × | 5.5 | hypospadias repair cryptorchidopexy | p.E367G (het) | *CST9*: p.R87* (het) |
| **I19** | 16y | M | √ | perineal | inguinal | intrascrotal | × | / | √ | abnormal spleen | 5.5 | × | p.S430I (het) | *HSD17B3*: p.I60T (het) *WT1*: p.T7P (het) |

**Abbreviations:** y, years; EMS, External Masculinization Score; F, female; M, male; FEG, female external genitalia; hom, homozygous; hemi, hemizygous; het, heterozygous.

**Supplementary Table 5. Laboratory features of the cohort**

| Variable (n) | n | % | Patient ID |
| --- | --- | --- | --- |
| Prepubertal patients (n=8) |  |  |  |
| hCG stimulation tests (n=8) |  |  |  |
| Normal testosterone synthesis | 4 | 50 | I8, I14, I15, I17 |
| Testosterone synthesis defects | 4 | 50 | I4, I5, I11, I16 |
| Pubertal and post-pubertal patients (n=7) |  |  |  |
| FSH (n=7) |  |  |  |
| High | 7 | 100 | I1, I7, I9, I10, I13, I18, I19 |
| Normal | 0 | 0 | - |
| Low | 0 | 0 | - |
| LH (n=7) |  |  |  |
| High | 5 | 71 | I1, I7, I9, I10, I13 |
| Normal | 2 | 29 | I18, I19 |
| Low | 0 | 0 | - |
| T (n=7) |  |  |  |
| High | 0 | 0 | - |
| Normal | 5 | 71 | I1, I7, I9, I13, I19 |
| Low | 2 | 29 | I10, I18 |
| All patients (n=15) |  |  |  |
| AMH (n=13) |  |  |  |
| High | 0 | 0 | - |
| Normal | 3 | 23 | I4, I8, I17 |
| Low | 10 | 77 | I1, I5, I7, I9, I10, I11, I13, I14, I16, I18 |
| ACTH (n=15) |  |  |  |
| High | 5 | 33 | I1, I5, I9, I14, I17 |
| Normal | 10 | 67 | I2, I3, I4, I8, I10, I13, I15, I16, I18, I19 |
| Low | 0 | 0 | - |
| Cortisol (n=15) |  |  |  |
| High | 0 | 0 | - |
| Normal | 4 | 27 | I3, I9, I10, I17 |
| Mild low* | 11 | 73 | I1, I2, I4, I5, I8, I13, I14, I15, I16, I18, I19 |
| Low | 0 | 0 | - |
| ACTH stimulation tests (n=6) |  |  |  |
| Normal adrenocortical function | 6 | 100 | I1, I2, I3, I4, I5, I9 |
| Adrenocortical function defects | 0 | 0 | - |
| 11-oxygenated androgens  (n=11, post-adrenarche age) |  |  |  |
| High or Normal | 4 | 36 | I3, I5, I6, I19 |
| Low (≥3 items) | 7 | 64 | I1, I2, I4, I9, I10, I13, I18 |

* Mild low indicates that the measured cortisol levels are within the range of 3–18 μg/dl (measured by CLIA method), necessitating an ACTH stimulation test for further evaluation of adrenal cortical function.

**Supplementary Table 6. Laboratory findings (sex hormone levels) of the cohort**

| **No.** | **Age** | **FSH（mIU/ml）** | **LH（mIU/ml）** | **T**  **(ng/ml)**  **(B/A)** | **PRL**  **(ng/ml)** | **AD**  **(ng/ml)**  **(B/A)** | **DHEA-S**  **(ng/ml)**  **(B/A)** | **F-T**  **(pg/ml)**  **(B/A)** | **DHT**  **(pg/ml)**  **(B/A)** | **17-OHP**  **(ng/ml)**  **(B/A)** | **AMH**  **(ng/ml)** | **SHBG**  **(nmol/L)** |
| --- | --- | --- | --- | --- | --- | --- | --- | --- | --- | --- | --- | --- |
| I1 | 15y | 46.39 | 26.29 | 3.85 | 65.06 | 1.89 | 2090 | 11.47 | 146.93 | 1.26 | 0.21 | NA |
| I2 † | 19y | 74.34 | 31.18 | 0.32 | 19.06 | 1.45 | 1800 | 1.12 | 41.16 | 0.47 | NA | 17.2 |
| I3 † | 24y | 75.66 | 16.12 | 0.28 | 5.93 | 1.37 | 642 | NA | NA | NA | <0.02 | NA |
| I4 ‡ | 10y | 2.27 | 0.19 | 0.13/0.96 | 6.27 | 0.38/0.79 | 720/703 | 1.75 | 21.82/61.02 | 0.19 | 159.1 | NA |
| I5 ‡ | 5y | 8.13 | 0.12 | 0.13/0.65 | 12.19 | <0.30/0.35 | <150/204 | 0.3 | 11.1/32.20 | 0.43 | 29.29 | 161.1 |
|  | 10y | 17.68 | 7.24 | 3.84 | 11.01 | 0.60 | 820.9 | NA | 0.15 | 0.48 | 7.38 | NA |
| I6 † | 16y | 44.51 | 19.54 | 0.25 | 13.9 | 0.84 | 1310 | NA | NA | NA | NA | NA |
| I7 | 22y | 63.88 | 37.06 | 1.83 | 25.8 | 4.91 | 684 | 5.12 | NA | NA | 0.13 | 40.48 |
| I8 ‡ | 1y | 2.6 | 0.84 | 1.23/6.98 | 21.83 | <0.30/0.49 | <150/<150 | 1.23/8.23 | 76.04/358.04 | 0.39 | 95.88 | >200 |
| I9 | 15y | 64.05 | 22.19 | 4.07 | 36.07 | 1.61 | <150 | NA | 141.87 | 1.05 | 0.21 | 61.3 |
| I10 | 22y | 67.6 | 30.66 | 0.15 | NA | 1.03 | 2432 | NA | 63.76 | 0.74 | 0.09 | NA |
| I11 ‡ | 5y | 2.71 | <0.07 | 0.08/0.16 | 17.16 | 0.58/0.60 | 712 | NA | 15.19/18.87 | 1.49 | 16.04 | NA |
| I12 | 21y | NA | NA | NA | NA | NA | NA | NA | NA | NA | NA | NA |
| I13 | 19y | 79.94 | 51.33 | 2.8 | 30.9 | 0.98 | 1861 | NA | 124.72 | 0.62 | 0.37 | NA |
| I14 ‡ | 3y | 6.25 | 0.6 | 0.21/3.32 | 6.59 | 0.29/0.31 | 132 | NA | 61.35/46.05 | 0.92/0.26 | 12.35 | NA |
| I15 ‡ | 4y | 4.18 | 0.23 | 0.09/5.53 | 14.4 | <0.3/<0.3 | 124 | NA | 20.48/482.95 | NA | NA | NA |
| I16 ‡ | 8y | 6.61 | 0.65 | 0.13/0.75 | 14.9 | 0.39/0.54 | 483/613 | NA | 29.99 | 0.88/0.56 | 17.81 | NA |
| I17 ‡ | 3y | 0.77 | 0.06 | 0.13/1.76 | 16.06 | 0.14/0.26 | 221 | NA | 6.96/71.32 | 0.33/0.36 | 57.6 | NA |
| I18 | 28y | 41.73 | 11.4 | 1.34 | NA | 1.77 | 2508 | NA | 180.21 | 0.9 | 0.11 | NA |
| I19 | 16y | 50.65 | 12.02 | 2.54 | NA | NA | NA | NA | NA | NA | NA | NA |

**Abbreviations:** FSH, Follicle-stimulating hormone; LH, Luteinizing hormone; T, Testosterone; PRL, prolactin; AD, Androstenedione; DHEA-S, Sulfated dehydroepiandrosterone; F-T, Free-testosterone; DHT, Dihydrotestosterone; 17-OHP, 17-Hydroxyprogesterone; AMH, Anti-müllerian hormone; SHBG, Sex hormone-binding globulin; B-before hCG stimulate; A-after hCG stimulate; y, years; NA, not available.

**Reference range:** FSH, 0.95-11.95mIU/ml; LH, 0.57-12.07mIU/ml; T, 1.42-9.23ng/ml; PRL, 3.46-19.4ng/ml; AD, 0.7-3.6ng/ml; DHEA-S, 800-5600ng/ml; F-T, 8.69-54.69pg/ml; DHT, 17.9-579pg/ml (male, 21-91y); 17-OHP, 0.5-2.4ng/ml; AMH. 38.79-294.19ng/ml(<11y), 2.06-100.02ng/ml(11-20y), 0.63-19.66ng/ml(>20y); SHBG, 14.5-48.4nmol/L (male, 17-65y), 26.1-110nmol/L (female, 17-50y), 14.1-68.9nmol/L (female, post-menopause). †, no testis/gonad or after orchiectomy; ‡, prepubertal patients.

**Supplementary Table 7.** **Laboratory findings (adrenal function-diurnal rhythms for ACTH and cortisol and ACTH stimulation test) of the cohort**

| **No.** | **Age** | **ACTH(pg/ml)**  **8am-4pm-0am** | **F(μg/dL)**  **8am-4pm-0am** | **After ACTH stimulate (0min-30min-60min)** | | | | | **24h UFC**  **(μg/24h)** |
| --- | --- | --- | --- | --- | --- | --- | --- | --- | --- |
|  |  |  |  | **F**  **(μg/dL)** | **P**  **(ng/ml)** | **17OHP**  **(ng/ml)** | **AD**  **(ng/ml)** | **DHEA-S**  **(ng/ml)** |  |
| I1 | 15y | 93.2-22.8-NA | 16.3-3.7-NA | 16.3-19.5-21.2 | 0.2-0.3-0.3 | 1.26-1.27-1.31 | 1.89-1.61-1.51 | 2090-1900-2200 | 49.84 |
| I2 † | 19y | 14.4 | 13.9 | 13.9-30.5-35.2 | 0.1-0.9-1.0 | 0.47-0.5-1.63 | 1.45-2.34-2.53 | 1800-1700-1780 | 21.84 |
| I3 † | 24y | 25.3 | 18.8 | 18.8-26.1-13.1 | 0.1-0.3-NA | NA | 0.82-1.53-1.69 | 642-661-581 | NA |
| I4 ‡ | 10y | 29.3 | 5.41 | 5.41-26.25-29.11 | 0.1-0.4-0.5 | 0.36-NA-0.84 | 0.38-NA-0.73 | 1379-NA-1556 | 262.24 |
| I5 ‡ | 10y | 54.0-29.4-17.4 | 10.59-3.27-6.00 | 10.59-20.39-21.73 | 0.1-0.4-0.4 | 0.48-NA-0.75 | 0.60-NA-0.77 | 820.9-NA-833.6 | NA |
| I6† | 16y | NA | NA | NA | 0.1 | NA | 0.84 | 1310 | NA |
| I7 | 22y | NA | NA | NA | 0.2 | NA | 4.91 | 684 | NA |
| I8‡ | 1y | 21.1 | 9.4 | 9.4 | 0.1 | 0.39 | <0.30 | <150 | NA |
| I9 | 15y | 62.7 | 19.3 | 19.3-24.2-23.1 | 0.3-0.3-0.4 | 1.05-1.50-1.59 | 1.61-1.71-1.64 | <150-210-175 | 27.37 |
| I10 | 22y | 41.3 | 20.7 | 20.7 | 0.2 | 0.74 | 1.03 | 2432 | 165.8 |
| I11‡ | 5y | NA | NA | NA | NA | 1.49 | 0.58 | 712 | NA |
| I12 | 21y | NA | NA | NA | NA | NA | NA | NA | NA |
| I13 | 19y | 26.6 | 15.4 | 15.4 | 0.1 | 0.62 | 0.98 | 1861 | NA |
| I14 ‡ | 3y | 49.2 | 11.6 | 11.6 | 0.1 | 0.92 | 0.29 | 132 | NA |
| I15 ‡ | 4y | 42.6 | 17.2 | 17.2 | 0.1 | NA | <0.3 | 124 | NA |
| I16‡ | 8y | 15.3 | 11.4 | 11.4 | 0.3 | 0.88 | 0.39 | 483 | NA |
| I17‡ | 3y | 64.7 | 31.3 | 31.3 | 0.1 | 0.33 | 0.14 | 221 | NA |
| I18 | 28y | 29.4 | 17 | 17 | 0.1 | 0.9 | 1.77 | 2508 | NA |
| I19 | 16y | 38.2 | 16.3 | 16.3 | 0.1 | NA | NA | NA | NA |

**Abbreviations:** ACTH, Adrenocorticotropic hormone; F, Cortisol; P, Progesterone; 17-OHP, 17-Hydroxyprogesterone; AD, Androstenedione; DHEA-S, Sulfated dehydroepiandrosterone; 24h UFC, 24 hours-Urinary free cortisol; y, years; NA, not available.

**Reference range:** ACTH, 0-46pg/ml; F, 5-25μg/dl; P, 0-0.2ng/ml; 17-OHP, 0.5-2.4ng/ml; AD, 0.7-3.6ng/ml; DHEA-S, 800-5600ng/ml (all age groups), 166-2427ng/ml (male, 11-14y), 451-3850ng/ml (male, 15-19y), 2384-5393ng/ml (male, 20-24y), 1679-5919ng/ml (male, 25-34y), 1397-4844ng/ml (male, 35-44y), 1362-4476ng/ml (male, 45-54y), 486-3618ng/ml (male, 55-64y), 2285-2836ng/ml (male, 65-70y); 24h UFC, 21-111μg/24h.

**Supplementary Table 8-1. Laboratory findings (LC-MS/MS) of the cohort**

| **No.** | **Age** |  | **T**  **(ng/ml)** | **DHT**  **(ng/ml)** | **AD**  **(ng/ml)** | **DHEA**  **(ng/ml)** | **DHEA-S**  **(ng/ml)** | **11KT**  **(nmol/L)** | **11OHT**  **(nmol/L)** | **11KA4**  **(nmol/L)** | **11OHA4**  **(nmol/L)** |
| --- | --- | --- | --- | --- | --- | --- | --- | --- | --- | --- | --- |
| I1 | 15y | ACTH-0 min | 3.172 | 0.089 | 0.651 | 4.473 | 2214.857 | 1.29 | 0.15 | 0.24 | 2.40 |
|  |  | ACTH-30 min | 2.595 | 0.083 | 0.627 | 4.619 | 2223.919 | 0.97 | 0.15 | 0.20 | 2.21 |
|  |  | ACTH-60 min | 2.623 | 0.079 | 0.583 | 4.455 | 2358.837 | 0.69 | 0.10 | 0.16 | 1.95 |
| I2 † | 19y | ACTH-0 min | 0.196 | 0.02 | 0.503 | 2.93 | 2030.872 | 0.59 | 0.11 | 0.17 | 1.23 |
|  |  | ACTH-30 min | 0.232 | 0.021 | 0.926 | 9.272 | 2130.178 | 0.55 | 0.20 | 0.16 | 2.28 |
|  |  | ACTH-60 min | 0.234 | 0.023 | 1.064 | 9.58 | 1977.36 | 0.28 | 0.15 | 0.08 | 3.19 |
| I3 † | 24y |  | 0.176 | 0.017 | 0.709 | 2.807 | 786.505 | 3.33 | 1.04 | 0.27 | 5.48 |
| I4 ‡ | 10y | Before hCG | 0.054 | 0.013 | 0.183 | 0.974 | 640.358 | 0.70 | 0.04 | 0.08 | 0.86 |
|  |  | After hCG | 0.93 | 0.065 | 0.377 | 2.821 | 719.593 | 0.71 | 0.11 | 0.06 | 1.60 |
| I5 ‡ | 5y | Before hCG | 0.041 | 0.007 | 0.17 | 0.316 | 111.182 | 3.08 | 0.06 | 0.40 | 1.39 |
|  |  | After hCG | 0.621 | 0.049 | 0.24 | 0.559 | 171.479 | 1.82 | 0.05 | 0.37 | 1.63 |
|  | 10y |  | 3.544 | 0.288 | 0.553 | 1.998 | 771.353 | 1.76 | 0.09 | 1.02 | 3.53 |
| I6 † | 16y |  | 0.145 | 0.057 | 1.082 | 4.008 | 702.354 | 1.86 | 0.59 | 1.04 | 4.30 |
| I7 | 22y |  | NA | NA | NA | NA | NA | NA | NA | NA | NA |
| I8 ‡ | 1y | Before hCG | 0.893 | 0.089 | 0.04 | 0.09 | 16.119 | 0.15 | ＜0.03 | 0.05 | 0.16 |
|  |  | After hCG | 6.246 | 0.46 | 0.197 | 0.116 | 51.278 | 0.04 | ＜0.03 | 0.02 | 0.06 |
| I9 | 15y |  | 3.098 | 0.146 | 0.687 | 0.815 | 102.533 | 1.89 | 0.27 | 0.17 | 2.64 |
| I10 | 22y |  | 0.117 | 0.047 | 0.409 | 7.193 | 1937.096 | 0.60 | 0.18 | 0.25 | 2.49 |
| I11 ‡ | 5y |  | NA | NA | NA | NA | NA | NA | NA | NA | NA |
| I12 | 21y |  | NA | NA | NA | NA | NA | NA | NA | NA | NA |
| I13 | 19y |  | 3.273 | 0.096 | 0.412 | 1.655 | 1274.205 | 0.66 | 0.12 | 0.10 | 1.44 |
| I14 ‡ | 3y |  | 2.961 | 0.393 | 0.092 | 0.065 | 25.044 | 0.31 | ＜0.03 | 0.01 | 0.33 |
| I15 ‡ | 4y |  | NA | NA | NA | NA | NA | NA | NA | NA | NA |
| I16 ‡ | 8y |  | 1.293 | 0.067 | 0.311 | 1.275 | 1250.668 | 0.54 | 0.01 | 0.10 | 1.09 |
| I17 ‡ | 3y |  | 0.004 | 0.105 | 0.037 | 0.021 | 80.102 | 0.26 | ＜0.03 | 0.09 | 0.41 |
| I18 | 28y |  | 9.173 | 0.409 | 1.427 | 2.99 | 548.515 | 0.77 | 0.13 | 0.37 | 2.80 |
| I19 | 16y |  | 3.736 | 0.176 | 1.444 | 7.942 | 2937.319 | 4.00 | 0.94 | 1.40 | 8.45 |

**Abbreviations:** T, Testosterone; DHT, Dihydrotestosterone; AD, Androstenedione; DHEA, Dehydroepiandrosterone; DHEA-S, Sulfated dehydroepiandrosterone; 11-KT, 11-ketotestosterone; 11OHT, 11β-hydroxytestosterone; 11KA4, 11-ketoandrostenedione; 11OHA4, 11β-hydroxyandrostenedione; y, years; NA, not available.

**Reference range:** T, 0.02-0.45ng/ml (female), 2.5-11ng/ml (male); DHT, <0.3ng/ml (female), 0.11-0.96ng/ml (male); AD, 0.30-2.00ng/ml; DHEA, <13ng/ml; DHEA-S, 280-3900ng/ml; 11KT, 1.0 (0.7-1.4) nmol/L; 11OHT, 0.4 (0.4-0.6) nmol/L; 11KA4, 0.6 (0.4-0.7) nmol/L; 11OHA4, 4.1 (3.1-5.6) nmol/L. The reference range of four 11-oxygenated androgens were shown as median (IQR) and sourced from the data of 69 men aged 18-39 (Davio *et al*. 2020).

**Supplementary Table 8-2. Laboratory findings (LC-MS/MS) of the cohort**

| **No.** | **Age** |  | **F**  **(ng/ml)** | **E1**  **(ng/ml)** | **E2**  **(ng/ml)** | **P**  **(ng/ml)** | **17-OHP**  **(ng/ml)** |
| --- | --- | --- | --- | --- | --- | --- | --- |
| I1 | 15y | ACTH-0 min | 89.093 | 0.013 | 0.024 | 0.111 | 0.576 |
|  |  | ACTH-30 min | 113.977 | 0.011 | 0.015 | 0.15 | 0.537 |
|  |  | ACTH-60 min | 121.663 | 0.009 | 0.018 | 0.17 | 0.514 |
| I2 † | 19y | ACTH-0 min | 74.301 | 0.01 | 0.013 | 0.049 | 0.173 |
|  |  | ACTH-30 min | 210.445 | 0.012 | 0.013 | 0.746 | 1.169 |
|  |  | ACTH-60 min | 240.201 | 0.011 | 0.014 | 0.951 | 1.154 |
| I3 † | 24y |  | 94.753 | 0.011 | 0.014 | 0.025 | 0.112 |
| I4 ‡ | 10y | Before hCG | 24.044 | 0.002 | 0.014 | 0.018 | 0.066 |
|  |  | After hCG | 150.931 | 0.004 | 0.014 | 0.085 | 0.395 |
| I5 ‡ | 5y | Before hCG | 111.707 | 0.001 | 0.013 | 0.032 | 0.251 |
|  |  | After hCG | 151.789 | 0.002 | 0.008 | 0.139 | 0.639 |
|  | 10y |  | 77.137 | 0.016 | 0.019 | 0.596 | 0.413 |
| I6 † | 16y |  | 79.992 | 0.196 | 0.04 | 0.109 | 0.309 |
| I7 | 22y |  | NA | NA | NA | NA | NA |
| I8 ‡ | 1y | Before hCG | 53.334 | <0.001 | 0.013 | 0.023 | 0.133 |
|  |  | After hCG | 63.331 | 0.001 | 0.013 | 0.004 | 0.369 |
| I9 | 15y |  | 116.268 | 0.01 | 0.014 | 0.092 | 0.772 |
| I10 | 22y |  | 142.656 | 0.029 | 0.014 | 0.095 | 0.176 |
| I11 ‡ | 5y |  | NA | NA | NA | NA | NA |
| I12 | 21y |  | NA | NA | NA | NA | NA |
| I13 | 19y |  | 73.662 | 0.011 | 0.024 | 0.024 | 0.292 |
| I14 ‡ | 3y |  | 79.078 | 0.001 | 0.011 | 0.039 | 0.256 |
| I15 ‡ | 4y |  | NA | NA | NA | NA | NA |
| I16 ‡ | 8y |  | 74.251 | 0.007 | 0.016 | 0.069 | 0.299 |
| I17 ‡ | 3y |  | 86.256 | <0.001 | 0.007 | 0.028 | 0.048 |
| I18 | 28y |  | 103.454 | 0.026 | 0.027 | 0.056 | 0.254 |
| I19 | 16y |  | 115.239 | 0.021 | 0.024 | 0.088 | 0.601 |

**Abbreviations:** F, Cortisol; E1, Estrone; E2, Estradiol; P, Progesterone; 17-OHP, 17-Hydroxyprogesterone; y, years; NA, not available. †, no testis/gonad or after orchiectomy; ‡, prepubertal patients.

**Reference range:** F, 60-260ng/ml; E1, <0.069ng/ml; E2, 0.15-0.35ng/ml (female), 0.01-0.04ng/ml (male); P, 3.0-31.4ng/ml (female), <0.2ng/ml (male); 17-OHP, <2.2ng/ml.

**Supplementary Table 9. Radiographic characteristics of the cohort**

| Variable (n) | n | % | Patient ID |
| --- | --- | --- | --- |
| Internal genitalia development (n=19) |  |  |  |
| Normal | 0 | 0 | - |
| Abnormal | 19 | 100 | All |
| Bone age (n=6) |  |  |  |
| Normal | 3 | 50 | I2, I4, I6 |
| Delayed | 3 | 50 | I3, I9, I10 |
| Adrenal development (n=6) |  |  |  |
| Normal | 5 | 83 | I1, I2, I4, I5, I19 |
| Abnormal | 1 | 17 | I9 |
| Spleen development (n=8) |  |  |  |
| Normal | 2 | 25 | I1, I3 |
| Abnormal | 6 | 75 | I2, I4, I5, I9, I17, I19 |

**References.**

1. Ito Y, Unagami M, Yamabe F, Mitsui Y, Nakajima K, Nagao K, et al. A method for utilizing automated machine learning for histopathological classification of testis based on Johnsen scores. Sci Rep. 2021;11(1):9962.
